# Pleiotropic Roles of P2X7 in the Central Nervous System

**DOI:** 10.3389/fncel.2019.00401

**Published:** 2019-09-04

**Authors:** Jean M. Kanellopoulos, Cécile Delarasse

**Affiliations:** ^1^CNRS, I2BC, Université Paris-Saclay, Orsay, France; ^2^Inserm, Sorbonne Université, CNRS, Institut de la Vision, Paris, France

**Keywords:** purinergic receptor, P2X7, ATP, nervous system, neurodegenerative disease, neurologic disease, demyelinating disease, animal model

## Abstract

The purinergic receptor P2X7 is expressed in neural and immune cells known to be involved in neurological diseases. Its ligand, ATP, is a signaling molecule that can act as a neurotransmitter in physiological conditions or as a danger signal when released in high amount by damaged/dying cells or activated glial cells. Thus, ATP is a danger-associated molecular pattern. Binding of ATP by P2X7 leads to the activation of different biochemical pathways, depending on the physiological or pathological environment. The aim of this review is to discuss various functions of P2X7 in the immune and central nervous systems. We present evidence that P2X7 may have a detrimental or beneficial role in the nervous system, in the context of neurological pathologies: epilepsy, Alzheimer’s disease, multiple sclerosis, amyotrophic lateral sclerosis, age-related macular degeneration and cerebral artery occlusion.

## Introduction

The “purinergic hypothesis” was introduced in Burnstock (1972), based on his studies showing that response to ATP was similar to the response of non-adrenergic, non-cholinergic nerve stimulation of gut or bladder smooth muscles. Another important concept was introduced when ATP was shown to be released with various neurotransmitters in the peripheral and central nervous systems (CNS; Burnstock et al., 1972). Burnstock’s hypothesis represents a major step in our understanding of purinergic signaling and Burnstock proposed pharmacological tools to discriminate between different receptor families for adenosine (P1 receptors) and for ATP/ADP (P2 receptors). P2-purinergic receptors are able to transduce signals triggered by the binding of extracellular adenosine 5′ triphosphate (eATP). However, eATP is rapidly degraded by several ecto-enzymes to adenosine 5′ diphosphate (ADP), adenosine 5′ monophosphate (AMP) and adenosine. ATP binds to plasma membrane receptors of the P2 family while adenosine stimulates other purinergic receptors called P1 receptors. The P2 family of receptors is divided in two subgroups, the ionotropic P2X receptors which bind ATP and metabotropic P2Y receptors able to bind ATP, ADP, UTP, and UDP.

The P2X7 receptor (P2X7) belongs to the P2X receptor family. The functions of P2X7 in inflammation and cell death have been studied extensively. The role of P2X7 in the immune system has been reviewed at length recently by Di Virgilio et al. (2017). In the CNS, the involvement of P2X7, in particular, in neuronal cell death is unclear, since the expression of P2X7 is still the subject of intense debate (Illes et al., 2017; Miras-Portugal et al., 2017). Interestingly, a recent in-depth study, using P2X7-transgenic reporter mice, cell-specific P2X7-deficient mice and P2X7-specific nanobodies (Kaczmarek-Hajek et al., 2018), showed that P2X7 is mainly expressed in glial cells. These important results are in agreement with a previous report that functional P2X7 are expressed on radial astrocytes of the cerebellar cortex called Bergmann cells (Habbas et al., 2011). However, using a humanized P2X7 conditional KO mouse line crossed to different tissue- or cell-specific Cre-recombinase transgenic mice, Metzger et al. (2017) have found that P2X7 mRNA is expressed in glutamatergic pyramidal neurons of the CA3 region of the hippocampus and in astrocytes, oligodendrocytes and microglia. Immune cells are involved in neurological diseases, such as multiple sclerosis in which an autoimmune response driven by T lymphocytes contributes to pathological processes. To a lesser extent, an inflammatory response also takes part in neurodegenerative diseases, as for example, in Alzheimer’s disease (AD), amyotrophic lateral sclerosis (ALS) and age-related macular degeneration (AMD). In these pathologies, danger signals like amyloid-β peptides and ATP are released which activate the innate immune system leading to the development of a sterile inflammation (Chen and Nunez, 2010). Thus, P2X7 activities vary and are modulated by the pathological environment, the amount of extracellular ATP, the cell types involved, the level of P2X7 expression as well as the expression of co-receptors (TLR, pannexin 1) (Di Virgilio et al., 2017). The potential therapeutic effects of P2X7 inhibition by antagonists in different animal models of neurological diseases have been elegantly reviewed by Bartlett et al. (2014). In the present review, we will provide an overview of works highlighting the pleiotropic functions of P2X7 in the CNS in physiological and pathological conditions.

## P2X7 Characteristics

### P2X7 Structure

Seven members of the P2X receptor family share the same predicted structure composed of a large extracellular loop of about 300 amino acids which binds ATP, two transmembrane domains, and two intracellular N- and C- termini. The P2X7 differs from other P2X receptors by its C terminus which is 200 amino acids longer. The first crystallographic structure of a P2X receptor was obtained by Kawate et al. (2009). It shows that the zebra fish P2X4 is organized as a trimer of P2X4 and a new fold named dolphin like was defined for each subunit. The same group published the structure of the ATP-bound to zebra fish P2X4 showing that two neighboring subunits were able to create a binding site for ATP and identified several residues involved in coordination of the phosphates of ATP as well as those interacting with adenine (Hattori and Gouaux, 2012). In addition, comparison of the free P2X4 with its ATP-bound form showed how the 6 transmembrane helices of the 3 subunits move from a closed to open state (Hattori and Gouaux, 2012). More recently, Karasawa and Kawate (2016) described the crystal structures of a truncated panda P2X7 in the presence of 5 different antagonists. They showed that the 5 drugs bind to the same pocket by hydrophobic interactions. This drug binding pocket is different from the ATP binding site (Karasawa and Kawate, 2016) and these antagonists behave as allosteric non-competitive inhibitors. Kasuya et al. (2017) have described the crystal structure of the chicken P2X7 in complex with the competitive inhibitor TNP-ATP. They found that the TNP-ATP molecule is positioned in the ATP binding pocket and identified the structural mechanism preventing channel activation.

### P2X7 Channel/Pore

Brief activation of P2X7 by ATP in its tetra-anionic form, ATP^4–^, opens cation-specific ion channels. Prolonged ligation of P2X7 results in the formation of non-selective membrane pores, permeable to molecules of molecular mass up to 900 Da. The molecular nature of this non-selective pore remains controversial. Two main hypotheses were proposed to explain pore formation, (1) P2X7 has the intrinsic ability to dilate and form the pore, (2) pore formation involves additional molecules such as plasma membrane hemichannels. The experimental evidence for or against the requirement of additional molecules to trigger the formation of the non-selective pore formation after P2X7 stimulation have been reviewed in detail by Di Virgilio et al. (2018). Several studies suggested that pannexin-1 (Pelegrin and Surprenant, 2006), connexin 43 (Beyer and Steinberg, 1991) and anoctamin 6 (Ousingsawat et al., 2015), a phospholipid scramblase, are involved in the formation of the non-selective pore. However, in mice deficient for pannexin-1 or connexin 43, the stimulation of P2X7 still triggers the formation of non-selective pores showing that these hemichannels of macrophages are dispensable (Qu et al., 2011; Alberto et al., 2013). Recently, purified panda P2X7 incorporated in proteoliposomes was able to form the non-selective pore after stimulation with ATP in the absence of other proteins. Thus, these findings strongly suggest that P2X7 possess an intrinsic ability to form a non-selective pore after ATP stimulation (Karasawa et al., 2017). In addition, a cysteine rich region containing C362 and C363 was demonstrated to be required for the formation of the non-selective pore. Mutations of these cysteine to serine eliminate YO-PRO-1 uptake. Palmitoylation of these cysteine plays a fundamental role in non-selective pore formation and it was proposed that these cysteine prevent the inhibitory effect of cholesterol (Karasawa et al., 2017). In addition, two elegant biochemical studies (Harkat et al., 2017; Pippel et al., 2017) strongly suggested that the non-selective pore formation is not due to a progressive dilation of the P2X7-channel letting large cations into cells. In contrast, ATP binding to P2X7 triggers the opening of a channel allowing the influx of small cations (Ca^2+^, Na^+^) and larger ones, such as YO-PRO1. Peverini et al. (2018) have recently analyzed and discussed current views on the permeability to large cations of P2X7 and other P2Xs following ATP stimulation in relation with the putative physiological roles of these non-selective pores.

However, several studies point out that other non-selective pores might be triggered by P2X7 stimulation. It was found that the non-selective pore formation was dependent of MAP-kinase activities (Donnelly-Roberts et al., 2004; Faria et al., 2005) and on second messengers such as Ca^2+^ (Faria et al., 2005). Faria et al. (2005) used a cell-attached configuration which allowed them to trigger or inhibit P2X7 located within the patch pipette or outside it on the plasma membrane. In peritoneal macrophages and 2BH4 cells, they found that a non-selective pore formation occurred both within the patch pipette and outside of the plasma membrane, after ATP stimulation in or outside the pipette. Importantly, ATP application outside the membrane patch lead to pore formation inside the patch pipette even after P2X7 had been blocked with the pharmacological inhibitor o-ATP within the pipette. These data lead them to postulate that P2X7 stimulation generates a second messenger diffusing inside the cell and triggering the formation of a non-selective pore (Faria et al., 2005).

In addition, Schachter et al. (2008) have compared fluorescent dye uptakes after ATP stimulation in HEK293-P2X7 and macrophages. They found that while cationic dye uptake increased in both cell types after P2X7 stimulation, only macrophages were able to take up anionic dye and to form the p440 pS channels (Schachter et al., 2008). These results suggest that P2X7 stimulation triggers two different pores in macrophages, one for cationic and another one for anionic dyes.

Karasawa et al. (2017) have shown that P2X7 activity, especially pore formation, was highly dependent on the lipid composition of the liposomes in which the purified panda P2X7 was incorporated. Increase in cholesterol induced a decrease in P2X7 non-selective pore formation while phosphatidylglycerol and sphingomyelin enhanced it (Karasawa et al., 2017). Another study reports that P2X7-non-selective pore formation is increased by diminution of plasma membrane cholesterol concentration in human and mouse cells (Robinson et al., 2014). Interestingly, lipin-2 deficient macrophages produce lower levels of cholesterol than WT macrophages, this decrease boosts P2X7 activity leading to an increase in non-selective pore formation, K^+^ efflux, NLRP3 activation and IL-1β/IL-18 release (Lorden et al., 2017). The physiopathological relevance of these observations is comforted by *in vivo* studies showing that LPS treatment of lipin-2ko mice induced highly significant increases in IL-1β and IL-18 serum levels compared to WT animals, an observation attributed in part to P2X7 hyperactivity (Lorden et al., 2017). Majeed syndrome patients have inactivating mutations of the *LPIN2* gene. The results obtained by Lorden et al. (2017) strongly suggest that auto-inflammatory disorders found in Majeed syndrome patients are due to an excessive production of mature IL-1β/IL-18 through P2X7 hyperactivity.

Several groups have reported that a pool of P2X7 is associated with detergent-resistant membranes (DRM) in different cell types (Garcia-Marcos et al., 2006; Barth et al., 2007; Delarasse et al., 2009; Gonnord et al., 2009) and that the two populations of P2X7 in the plasma membrane are associated with distinct receptor properties (Garcia-Marcos et al., 2006). Interestingly, the studies of Garcia-Marcos et al. (2006) indicate that the P2X7 involved in non-selective pore formation were those located outside the DRM, in membrane regions containing much less cholesterol.

Gonnord et al. (2009) have shown that P2X7 association with DRM requires the post-translational modification by palmitic acid of several P2X7 carboxy-terminal cysteins. Four regions of the carboxy terminus domain are involved in palmitoylation. Palmitoylation-defective P2X7 mutants showed a dramatic decrease of P2X7 cell surface expression due to their retention in the endoplasmic reticulum and proteolytic degradation in lysosomes and proteasomes. Thus, P2X7 palmitoylation plays a critical role in its association with the lipid microdomains of the plasma membrane and in the regulation of its half-life.

### P2X7 Induced Cell Death or Proliferation

**FIGURE 1:**
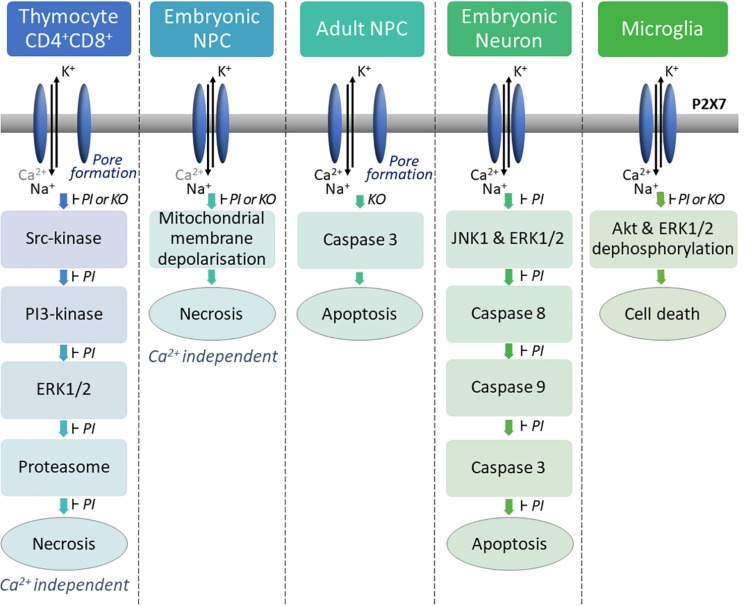
P2X7 activation induces cell death via different molecular pathways depending on the cell type. In mouse thymocytes, P2X7 stimulation induces P2X7-dependent pore formation and a calcium-independent cell lysis via the successive activation of a SRC family tyrosine kinase, a phosphatidylinositol 3-kinase, the mitogen-activated protein (MAP) kinase (ERK/2) module, and the proteasome (Auger et al., 2005). In embryonic mouse NPCs, activation of P2X7 does not lead to pore formation but induces mitochondrial membrane depolarization leading to calcium-independent lysis of the cell (Delarasse et al., 2009). High concentration of ATP induces P2X7-dependent pore formation in mouse adult NPCs and activation of caspase 3 leading to cell death (Messemer et al., 2013). P2X7 stimulation induces apoptosis in embryonic rat cortical neurons *via* activation of the signaling pathways JNK1, ERK and caspases 8/9/3 (Kong et al., 2005). In newborn mouse microglia, P2X7 stimulation induces dephosphorylation of AKT, ERK1/2 and cell death (He et al., 2017). PI: Pharmacological inhibitors were used to specifically inhibit P2X7 or each down-stream enzyme of the pathway. Each of them were able to block P2X7-dependent cell death. KO: Cells from P2X7ko mice were used to analyze P2X7-dependent cell death pathway compared to cells from WT mice.

Prolonged ATP stimulation of P2X7 can lead to membrane blebbing and cell death by apoptosis or lysis/necrosis depending on the cell type. P2X7 is expressed by various hematopoietic cells such as thymocytes, lymphocytes, macrophages, dendritic cells. ATP treatment of mouse thymocytes induces two types of P2X7-dependent cell death: (1) a caspase-dependent apoptosis of a small subpopulation of thymocytes (mostly CD4^+^); (2) a predominant Ca^2+^ independent lysis/necrosis of CD4^+^CD8^+^ thymocytes (Auger et al., 2005). Our analyses of the biochemical pathways triggered after ATP stimulation of thymocyte have shown that P2X7 ligation induces the sequential activation of a Src-kinase, a PI3-kinase (phosphoinositide 3-kinase), the ERK1/2 kinases (extracellular signal-regulated kinases) and finally the proteasome (Figure 1). Importantly, the pharmacologic inhibition of one of these enzymatic activities blocks the P2X7-induced thymocyte lysis. Surprisingly, we were unable to demonstrate these biochemical pathways of cellular death in T splenocytes (unpublished observations). The reason of this discrepancy may lie in the recent findings that P2X7-mediated cellular activities in heterogeneous sub-populations of T lymphocytes in the spleen are not directly related to the levels of P2X7 membrane expression but depend on the stage of activation/differentiation of these T cell subsets (Safya et al., 2018; Mellouk and Bobe, 2019).

In Figure 1, several examples of P2X7 induced cell death are shown. Various biochemical pathways are activated in different cell types undergoing necrosis/lysis, preventing the identification of a characteristic P2X7-induced cell death pathway. While necrotic/lysis induced by P2X7 stimulation remains ill defined, P2X7-dependent apoptosis and pyroptosis have been described more precisely at the molecular level. In particular, major breakthroughs have been made in our understanding of pyroptosis following P2X7-induced activation of NLRP3 inflammasome (cf. following paragraph).

While P2X7-dependent cell deaths have been described in many cellular models, the role of P2X7 in cell growth was discovered by Baricordi et al. (1999) who found that two different P2X7 negative lymphoid cell lines, transfected with a cDNA encoding P2X7, were able to grow in serum free medium. This increased proliferation was shown to be blocked by the antagonist o-ATP and was due to the release of ATP in the culture supernatants of these leukemic cell lines (Baricordi et al., 1999). More recently, basal stimulation of P2X7 was shown to increase the mitochondrial potential ΔΨ, the mitochondrial Ca^2+^ concentration and stimulate ATP synthesis (Adinolfi et al., 2005). The increase in cytosolic Ca^2+^ concentrations following basal stimulation of P2X7 was shown to increase the amount of nuclear NFATc1 (nuclear factor of activated T cell complex 1) leading to cell growth (Adinolfi et al., 2009). Surprisingly, the basal stimulation of P2X7 leading to cell growth was dependent on the carboxy-terminal tail of P2X7 and the non-selective pore activity (Adinolfi et al., 2005).

Further studies have shown that the T-cell receptor stimulation of T lymphocytes triggers the release of ATP which plays a crucial role in increasing cytosolic Ca^2+^ concentration, NFAT activation and IL-2 secretion (Yip et al., 2009). Additional evidence suggesting that P2X7 may trigger T cell growth or promote survival was provided by Adinolfi et al. (2010) They identified a shorter P2X7 natural splice variant [P2X7(B)] which lacks the carboxy-terminal tail and is expressed in T lymphocytes. P2X7(B) transfected in HEK293 cells was expressed as an homotrimer at the plasma membrane and had many properties of the longer isoform P2X(A) but was unable to form the non-selective pore. Interestingly, when P2X7(A) and P2X7(B) were co-transfected into HEK293 cells, they were able to form heterotrimers expressed at the plasma membrane and which have different properties. Their results suggest that if P2X7(A) is predominant in the heterotrimer, P2X7 will trigger the opening of the non-selective pore leading to cell death. In contrast, if the shorter isoform is in excess, P2X7 will stimulate growth (Adinolfi et al., 2010). However, the existence and functions of P2X7(A)/P2X7(B) heterotrimers in T lymphocytes is lacking at present.

Thus, P2X7 stimulation can lead to opposite effects: cell death or cell growth. The concentration of ATP used to trigger P2X7 bearing cells may explain these dramatically opposite outcomes. The studies of Stojilkovic’s group (Khadra et al., 2013) have shown that naive P2X7 is activated and deactivated monophasically at low agonist concentrations, while at high concentrations P2X7 is activated with increased current amplitude and slow deactivation. They proposed a model explaining their data: at low agonist concentrations, only two ATP binding sites of the trimeric P2X7 are engaged and the channel opens to a low conductance state. Conversely, at higher agonist concentrations, all 3 binding sites are filled and the channel pore is dilated to a high conductance state.

### Activation of the NLRP3 Inflammasome

**FIGURE 2:**
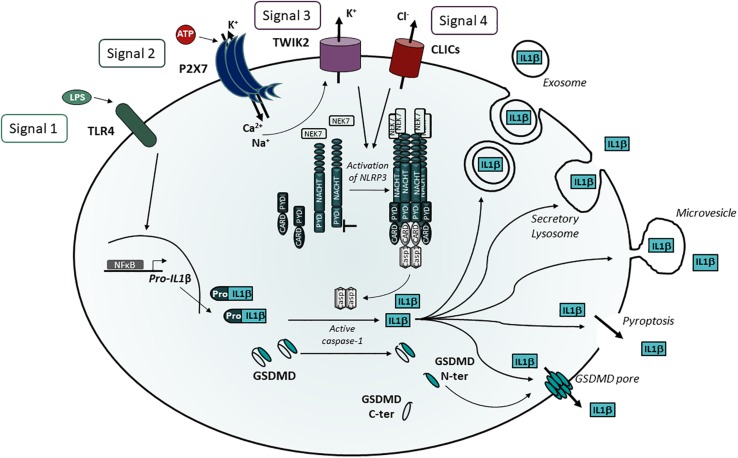
P2X7 stimulation induces NLRP3 activation leading to IL-1β release. Maturation of IL-1β requires 4 signals: 1) TLR activation triggering pro-IL-1β transcription, 2) Ca^2+^ and Na^+^ influxes via P2X7 stimulation leading to activation of TWIK2, 3) K^+^ efflux via P2X7 and TWIK2 leading to NEK7 binding to leucine rich domain of NLRP3, 4) Potential translocation of CLICs to the plasma membrane and Cl^–^ efflux and activation of NLRP3. NLRP3 oligomerization recruits ASC and induces pro-caspase 1 cleavage leading to the cleavage of pro-IL-1β in mature IL-1β. Several pathways have been described for IL-1β release via exosomes, secretory lysosomes and microvesicles. However, the identification of GSDMD as a substrate of caspase 1 unraveled the role of the GSDMD-N domain in pore formation and pyroptotic cell death.

The role of P2X7 in the processing and release of IL-1β and IL-18 by microglia and macrophages is well established (Ferrari et al., 1997a, b; Di Virgilio, 2007). The maturation and release of these interleukins require four signals (Figure 2). The first signal via Toll-like receptors drives pro-IL-1β transcription and accumulation in the cytosol, while pro-IL-18 is constitutively expressed. The second signal via P2X7 triggers the influxes of Ca^2+^ and Na^+^ and the membrane efflux of K^+^ (Steinberg et al., 1987; Riedel et al., 2007) via P2X7 itself but also via TWIK2 (two-pore domain weak inwardly rectifying K^+^ channel 2), a K^+^ channel belonging to the K2P family (Di et al., 2018). The third signal corresponds to the K^+^ efflux via P2X7 and TWIK2 which is a potent activator of the NLRP3 inflammasome. However, TWIK2 does not control the activation of AIM2, NLRC4 and pyrin inflammasomes (Di et al., 2018). TWIK2 may act in synergy with P2X7 which was considered to be a K^+^ channel (Steinberg et al., 1987; Riedel et al., 2007) because in the absence of TWIK2, K^+^ efflux was strongly decreased but not abolished (Di et al., 2018). Surprisingly, in P2X7ko macrophages, ATP was still able to trigger K^+^ efflux through TWIK2 suggesting that an undefined ATP-sensitive receptor is able to stimulate TWIK2. Thus, more work is required to identify the signal generated by P2X7 to trigger TWIK2 and activate NLRP3. The mechanism by which a decrease in cytosolic K^+^ concentration leads to NLRP3 activation is not yet fully understood. However, studies have recently identified NEK7, a member of the NIMA (never in mitosis gene a)-related serine/threonine kinase family, as a component of the NLRP3 inflammasome activation (He et al., 2016; Schmid-Burgk et al., 2016; Shi et al., 2016). NEK7 was important for NLRP3 activation and formed molecular complexes with NLRP3 by the interaction of its catalytic region with the NLRP3 leucine rich repeat domain independently of its kinase activity (He et al., 2016; Shi et al., 2016). Importantly, He et al. (2016) established that the presence of high concentration of extracellular KCl (50 mM), known to block K^+^ effluxes, inhibited the interaction of NLRP3 with NEK7 in ATP-stimulated macrophages. These experiments suggest that NEK7 is a sensor of K^+^. Recently, Sharif et al. (2019) described a cryo-electron microscopy structure of the human NLRP3 complexed to NEK7. Their interesting results show that the first half of the NEK7 C-lobe interacts with the leucin rich repeat of NLRP3 while the second part of the NEK7 C-lobe contacts the Nucleotide Binding Domain and the Helical Domain 2 of the NACHT region of NLRP3. The interaction between NLRP3 and NEK7 generates a complex which is inactive even after addition of ATP or ATP analogs. Thus, these ligands do not trigger oligomerization of NLRP3-NEK7 complexes *in vitro* and the stimulus required for NLRP3-NEK7 inflammasome formation remains undefined yet. Interestingly, another protein, the thioredoxin interacting protein (TXNIP) able to interact with NLRP3 was identified by the yeast two-hybrid method using the leucine-rich repeats of NLRP3 as bait (Zhou et al., 2010). In the cytosol, TXNIP and thioredoxin, an antioxidant component, form a complex which dissociates in the presence of ROS. Subsequently, TXNIP interacts with NLRP3 and activates the NLRP3 inflammasome. Many structurally unrelated NLRP3 inflammasome activators among which silica, alum, monosodium urate crystals and ATP are able to activate NLRP3, most probably because they induce the production of ROS and the formation of TXNIP-NLRP3 complexes. To the best of our knowledge, it is not known yet whether the NEK7-NLRP3 or TXNIP-NLRP3 complexes co-exist in the same cells or whether they are triggered in different cell types.

The 4th signal results from the translocation of the Cl^–^ intracellular channels (CLICs) to the plasma membrane as described below (Figure 2).

Notably, inhibition of NLRP3 activation and IL-1β release by high extracellular concentrations of KCl is due to the blockade of both K^+^ and Cl^–^ effluxes (Domingo-Fernandez et al., 2017; Tang et al., 2017; Green et al., 2018). Indeed, different pharmacological inhibitors of Cl^–^ channels prevent the secretion of IL-1β after stimulation of bone marrow derived macrophages (BMDM) by ATP (Domingo-Fernandez et al., 2017; Green et al., 2018). Importantly, it was established that the decrease in Cl^–^ effluxes induced a diminution of ASC specks formation in BMDM stimulated by LPS and ATP (Domingo-Fernandez et al., 2017; Tang et al., 2017; Green et al., 2018). The CLICs were shown to be involved in Cl^–^ effluxes and the knockdown by siRNA of CLIC1 and CLIC4 (Domingo-Fernandez et al., 2017) induced a strong diminution of pro-IL-1β and a total inhibition of mature IL-1β in the supernatant of LPS- and ATP- stimulated BMDM (Domingo-Fernandez et al., 2017). Since CLICS have redundant functions, another study used BMDM from CLIC1ko, CLIC4ko or CLIC5Ko mouse lines treated with siRNA against the other two Clic genes (Tang et al., 2017). Results of this strategy indicate that inhibition of CLIC1, CLIC4 and CLIC5 diminished ATP- and nigericin-induced caspase 1 maturation and IL-1β production and, in addition, established that CLICs activate NLRP3 by triggering Cl^–^ effluxes (Tang et al., 2017). Taken together these findings suggest that CLICs are acting downstream of K^+^ effluxes, mitochondrial damage and ROS production which are also stimulating NLRP3 activation after ATP- or nigericin-treatment of BMDM (Tang et al., 2017). More recently, Green et al. (2018) found that ASC speck formation is dependent of Cl^–^ effluxes and is reversible but this pathway does not trigger the activation of caspase 1 and IL-1β secretion unless there is a simultaneous efflux of K^+^ which stimulates the association of NEK7 to NLRP3. Thus, CLICs translocation to the plasma membrane and Cl^–^ efflux might be considered as a 4^th^ signal of NLRP3 activation.

NLRP3 is a large multimeric protein platform involved in the proteolytic cleavage of leaderless pro-IL-1β and pro-IL-18 to their active form by caspase 1. The activation of caspase 1 results from the binding of pro-caspase 1 by its caspase recruitment domain (CARD) to multimeric complexes composed of the Inflammasome NLRP3 and an adaptator called ASC (Apoptosis-associated speck like protein). ASC binds to the N-terminal pyrin domain (PYD) of NLRP3 by homotypic interaction via its PYD domain and recruits pro-caspase 1 by homotypic interactions of the CARD domains (Figure 2). The mature cytokines are then released from the cells. However, several pathways of secretion of mature cytokines have been described (Andrei et al., 1999; MacKenzie et al., 2001; Qu et al., 2007), and discussed (Lopez-Castejon and Brough, 2011). In LPS-activated human monocytes stimulated with exogenous ATP, Andrei et al. (1999) showed that mature IL-1β, active caspase 1 and cathepsin D co-localize in Lamp-1^+^ endolysosomes which fuse with the plasma membrane and release these products in the extra-cellular medium. They also found that phosphatidylcholine-specific phospholipase C and calcium-dependent phospholipase A2 were required for lysosomal exocytosis (Andrei et al., 2004). Another pathway of mature IL-1β release was described in THP1, a human monocytic cell line. P2X7 activation induced the release of phosphatidyl serine positive microvesicles containing mature and immature IL-1β and active caspase 1 (MacKenzie et al., 2001). Using cultures of primary bone marrow-derived macrophages, a third pathway of IL-1β release was described, in which P2X7 activation leads to the production of multivesicular bodies containing exosomes loaded with IL-1β, caspase 1 and NLRP3 inflammasome components (Qu et al., 2007; Qu and Dubyak, 2009). Recently, two groups have shown that mouse caspases 1 and 11 or human caspases 4 and 5 are able to cleave the cytosolic protein gasdermin D (GSDMD; Kayagaki et al., 2015; Shi et al., 2015). After proteolytic cleavage, the NH_2_-terminal fragment of GSDMD oligomerizes and forms pores in the cell plasma membrane (Ding et al., 2016; Liu et al., 2016; Sborgi et al., 2016). This leads to the release of inflammatory cytokines and pyroptotic cell death (Aglietti et al., 2016; Ding et al., 2016; Liu et al., 2016; Sborgi et al., 2016). In addition, several studies have suggested that the release of mature IL-1β is restricted to lytic macrophages (Liu et al., 2014; Cullen et al., 2015) and requires the lysis of macrophage plasma membrane. However, Evavold et al. (2018) and Heilig et al. (2018) have shown that blocking lysis of immortalized BMDM after NLRP3 inflammasome stimulation does not abolish the release of bioactive IL-1 which is then secreted through GSDMD pores. Interestingly, Ruhl et al. (2018) have recently disclosed that the endosomal sorting complexes required for transport (ESCRT) machinery is able to down-modulate GSDMD mediated pyroptosis and IL-1β secretion. These studies suggest that ESCRT-promoted ectosomes restore the injured plasma membranes by shedding GSDMD pores. This mechanism allows a GSDMD-mediated IL-1β release without cell lysis (reviewed in Evavold and Kagan, 2018). However, ATP (Evavold et al., 2018) and nigericin (Evavold et al., 2018; Heilig et al., 2018) triggered GSDMD-dependent pyroptosis preferentially while more potent NLRP3 activators (such as bacterial products or host-derived oxidized lipids) stimulated IL-1β release through GSDMD pores in absence of phagocyte lysis (Zanoni et al., 2017). In summary, following TLR activation of macrophages, P2X7 stimulation may trigger one of 4 different identified biochemical pathways leading to IL-1β secretion i.e., (i) fusion of IL-1β containing endolysosomes with plasma membrane, (ii) release of microvesicles, (iii) release of exosomes filled with mature IL-1β, or (iv) IL-1β release through GSDMD cell lysis. However, additional work is needed to define in which cells and how P2X7 stimulation preferentially triggers one of these pathways. The study of NLRP3 positive cells lacking or expressing low levels of GSDMD in physiological conditions may uncover which IL-1β release pathway is triggered.

The finding that TWIK2 is involved in NLRP3 activation and processing of IL-1β, raises the question of studying TWIK2 impact in cell death. Indeed, TWIK2-deficient BMDMs show decreased ATP-induced cell death without affecting nigericin cell cytotoxicity. Thus, an interesting question is whether TWIK2 is implicated in the various P2X7-dependent cell deaths (Figure 1) or whether members of the K2P family other than TWIK2 have a role in cell death of different cell types.

## P2X7 in the Nervous System

### Physiological Roles of P2X7 in the Nervous System

**FIGURE 3:**
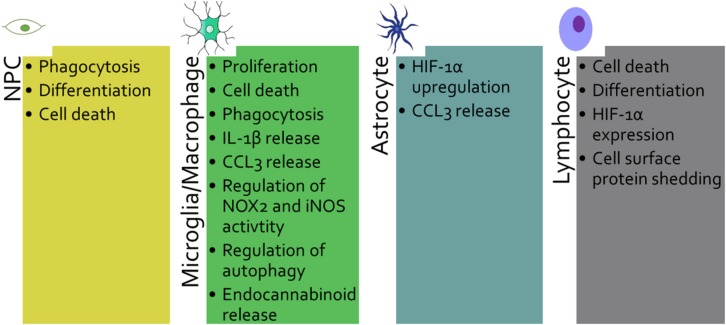
P2X7 plays different roles depending on the cell type that expresses it: Neural progenitor cell (NPC), microglia/macrophage, astrocyte and lymphocyte. The various functions of P2X7 are listed for each cell type.

**FIGURE 4:**
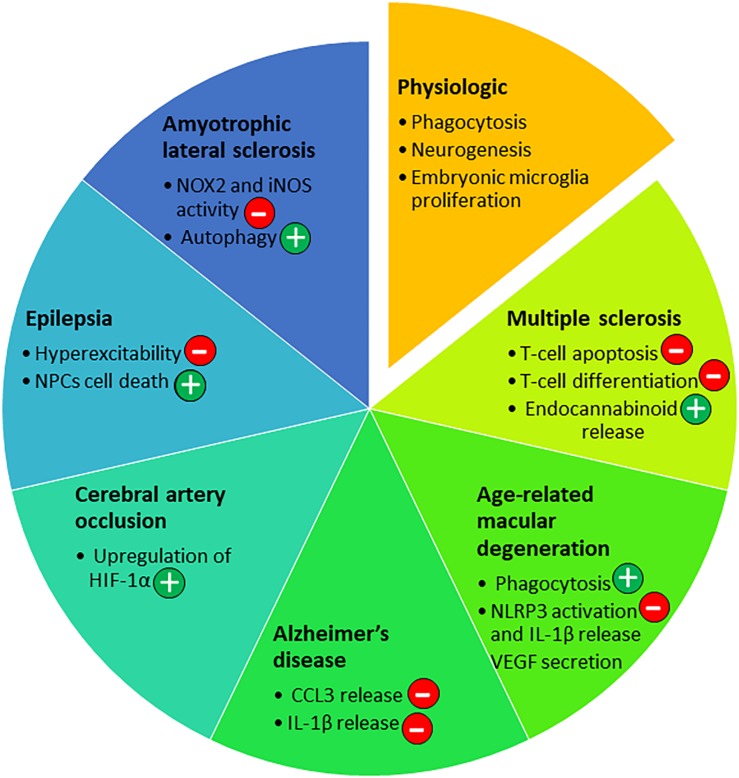
P2X7 roles in the nervous system in physiological and pathological conditions. Depending on the disease of the nervous system and the progression of the pathology, P2X7 has been involved in different pathways [beneficial (+) or not (*–*)] that are listed in the diagram.

Neural progenitor cells (NPCs) express functional P2X7 as shown by patch-clamp recording on mouse primary NPCs culture (Delarasse et al., 2009; Messemer et al., 2013) and hippocampal brain slice from nestin-EGFP mice (Messemer et al., 2013). P2X7 was reported to induce cell death and also proliferation. In mouse embryonic NPCs, P2X7 activation with high concentrations of ATP or the agonist Bz-ATP induces necrosis along with impaired mitochondrial function, as evidenced by the loss of mitochondrial membrane potential (Delarasse et al., 2009). In these cells, P2X7-dependent cell death occurred in the absence of P2X7 pore formation (Delarasse et al., 2009). In contrast, in mouse adult NPCs, P2X7 stimulation with high amount of Bz-ATP induces pore formation, activation of the pro-apoptotic caspase 3 and cell death (Messemer et al., 2013; Figure 1). In embryonic rat cortical neurons, activation of P2X7 with the same range of Bz-ATP concentrations leads to apoptosis via activation of JNK1 (c-jun N-terminal kinase 1), ERK1/2 and caspase 8/9/3 pathway (Kong et al., 2005). Thus, P2X7-dependent signaling pathways appear to change with the developmental status of the cells. In addition, P2X7 stimulation with low amounts of Bz-ATP in rat embryonic NPCs induced neuronal differentiation rather than cell death and pore formation (Tsao et al., 2013). P2X7 properties of adult mouse NPCs depend on the presence of exogenous ATP and on its concentration (Leeson et al., 2018). When ATP concentration is low, NPCs are in resting state, whereas high concentration of ATP brings about inflammatory conditions (Leeson et al., 2018). Three different functions have been highlighted: (1) phagocytosis in the absence of ATP, (2) calcium influx and a decrease in proliferation in the presence of low amounts of ATP and (3) pore formation leading to cell death in response to high amount of ATP. Interestingly, in the absence of serum, P2X7 expressed by human NPCs and neuroblasts could participate in the clearance of apoptotic neuronal cells, as a scavenger receptor (Lovelace et al., 2015). Overall, these data indicate that in inflammatory conditions, high amount of ATP is released and P2X7 contributes to detrimental processes by inducing NPCs cell death (Figures 3, 4). During embryonic development, P2X7 may be involved in the regulation of NPC population via phagocytosis of dead cells and proliferation of NPCs. The release of ATP from neighboring cells at a specific developmental stage and in a specific brain region may stop NPCs proliferation and induces NPC differentiation, thus P2X7 may also participate in neurogenesis (Figures 3, 4).

Microglia is the resident immune cell of the CNS, and shares many functions with macrophages such as phagocytosis, pro-inflammatory cytokines release and production of reactive oxygen species (ROS) and nitric oxide (NO). P2X7 was shown to activate microglia and to induce their proliferation (Bianco et al., 2006; Monif et al., 2009). Down-regulation of P2X7 by siRNA oligonucleotide in rat primary microglia decreased cell proliferation (Bianco et al., 2006). On the other hand, overexpression of P2X7 on microglia in neuron-glia mixed cell cultures from rat hippocampus leads to an increased number of microglial cells (Monif et al., 2009). In both models, P2X7-dependent proliferation was observed, in the absence of added exogenous P2X7 agonist, indicating that ATP released by neighboring astrocytes and microglia was sufficient to induce proliferation under resting conditions (Bianco et al., 2006; Monif et al., 2009). Evidence that the trophic effect of P2X7 is mediated by the non-selective pore activity was obtained by using a point mutant of P2X7 (P2X7 G345Y) that can form an intact functional channel but is defective in non-selective pore formation. Transfection of rat microglia with this mutated P2X7 prevented cell growth (Monif et al., 2009). This confirms the involvement of the non-selective pore in cell proliferation, previously demonstrated in P2X7-transfected HEK293 cells (Adinolfi et al., 2005). *In vivo*, in the embryonic spinal cord of mouse at E13.5 stage, Rigato et al. (2012) also showed that P2X7 controls proliferation of microglia, but not their activation state by comparing WT and P2X7ko mice. In contrast, in microglia from the cortex of newborn mice, prolonged stimulation of P2X7 with high amount of Bz-ATP induced cell death that may be induced via dephosphorylation of Akt and ERK (He et al., 2017; Figure 1). Thus, in the presence of physiological amounts of ATP, P2X7 may control microglia proliferation in the CNS while sustained activation may induce cell death (Figures 3, 4). Most studies have evaluated the death of cells on which P2X7 is activated by ATP. However, Skaper et al. (2006) have tested the impact of P2X7 stimulation of microglia on neighboring neurons. Co-cultures of rat cortical neurons with cortical microglia in the presence of ATP or Bz-ATP induced cortical neuron death. This effect was due to the release of superoxide and nitric oxide by P2X7 positive microglia stimulated with ATP and did not require direct contact between neuron and microglia. Thus, inhibition of P2X7 on microglia may protect neurons in some neurodegenerative diseases.

Age-related macular degeneration (AMD) is characterized by damage of the macula and central retina and the presence of extracellular deposits called drusen. Drusen are localized between the retinal pigment epithelium (RPE) and the Bruch’s membrane (BM) accompanied with accumulation of macrophages (Guillonneau et al., 2017). In the absence of ATP, it was clearly established that P2X7 acts as a scavenger receptor conferring phagocytic properties to murine peritoneal macrophages, human monocytes, macrophages and microglia in the absence of serum (Gu et al., 2011; Gu and Wiley, 2018; Janks et al., 2018). Co-inheritance of P2X7 G150R and P2X4 Y315C polymorphisms was shown to impair phagocytosis of particles by monocytes harboring these variants and to be associated with late-stage AMD, suggesting an involvement of P2X7 in pathological processes (Gu et al., 2013). In addition, 18-month old P2X7-deficient mice display BM thickening, RPE cell loss and increased number of microglia/macrophages in the subretinal space compared to age-matched WT mice (Vessey et al., 2017). These findings suggest that default in P2X7-dependent phagocytic properties may be involved in accumulation of drusen deposits and the development of AMD (Figures 3, 4).

### P2X7 Roles in Neurodegenerative Processes

Up-regulated expression of P2X7 has been observed in several neurodegenerative diseases: e.g., in astrocytes and microglia in AD (McLarnon et al., 2006; Martin et al., 2019), in microglia from spinal cord of MS and ALS (Yiangou et al., 2006), in astrocytes in MS (Narcisse et al., 2005; Amadio et al., 2017), in the optic nerve of MS patients (Matute et al., 2007) and in the neocortex and hippocampus of patients with epilepsy (Barros-Barbosa et al., 2016; Jimenez-Pacheco et al., 2016).

Epilepsy is a CNS disorder characterized by abnormal neuronal activity causing seizures. In mouse models, P2X7-deficient compared to WT animals show an increased susceptibility to pilocarpine-induced seizures. Pilocarpine activates muscarinic receptors leading to intracellular calcium release via IP3 production. In the absence of P2X7, desensitization of the muscarinic M1 receptor and pannexin 1 opening cannot be sustained, resulting in neuronal hyperexcitability involved in seizures (Kim and Kang, 2011). In addition, Rozmer et al. (2017) hypothesized that in the pilocarpine model, P2Y1 activation induced proliferation and migration of NPCs at ectopic site while P2X7 activation may counterbalance this pathologic effect by inducing the cell death of excess NPCs (Figures 3, 4). In contrast, no difference between P2X7-deficient and WT mice was observed in the seizures induced either by kainic acid which activates glutamate receptors, and picrotoxin which inhibits GABAA receptors (Kim and Kang, 2011). Surprisingly, in the kainic acid-induced seizure model, inhibition by pharmacological inhibitors of P2X7 (A438079 & JNJ-47965567) reduced seizures and gliosis (Engel et al., 2012; Jimenez-Pacheco et al., 2016; Beamer et al., 2017). This apparent discrepancy between results obtained with P2X7 selective pharmacological inhibitors and results found in the P2X7ko mice, may be explained by the level of P2X7 activation and the time at which P2X7 antagonists were administered. A constitutive deficit in P2X7 highlighted its physiological functions in the maintenance of the nervous system integrity. In the pilocarpine animal model of epilepsy, the study of P2X7-deficiency showed its potential roles in the control of the activity of neurons that express M1 receptors and in the regulation of neuronal networks by regulating NPCs population. Since pharmacological inhibitors were mostly applied during the acute phase of the kainic acid-induced disease, their effects suggest that at this later stage, high amounts of ATP are released and P2X7 present different properties i.e., release of IL-1β (Engel et al., 2012) and microglia proliferation in the hippocampus (Jimenez-Pacheco et al., 2016), which may contribute to pathological processes. Overall, these works illustrate that, depending on the model used and the pathological pathways involved, P2X7 may have a dual role i.e., P2X7 has protective role in physiological conditions while when the disease is advanced it worsens the pathological processes (Rassendren and Audinat, 2016).

In two independent studies, the degree of severity after middle cerebral artery occlusion (MCAO) was similar in WT and P2X7ko mice, indicating that P2X7 is not a major mediator of neuronal cell death in this model (Le Feuvre et al., 2003; Hirayama et al., 2015). Interestingly, a brief episode of sublethal ischemia protects neurons from cerebral ischemia, and this effect of ischemic tolerance is abolished in P2X7ko mice (Hirayama et al., 2015). These authors showed that P2X7 is up-regulated specifically in astrocytes but not in microglia during the tolerance episode and activation of P2X7 on astrocytes induces the transcription factor hypoxia inducible factor-1α (HIF-1α) upregulation and its target molecule erythropoietin, two neuroprotective factors. This study highlights a beneficial role of P2X7 up-regulation by astrocytes in ischemic tolerance and suggests that P2X7 may also have a protective effect in neurodegenerative diseases by inducing HIF-1α expression (Figures 3, 4).

Amyotrophic lateral sclerosis (ALS) is a fatal neurodegenerative disease characterized by the progressive death of motor neurons in the brain and spinal cord (Taylor et al., 2016). In ALS mouse model (SOD1-G93A), the lack of P2X7 leads to an increased severity of the disease (diminished motor performance and amplified motoneuron loss) accompanied by an increased astrogliosis and microglial reaction with upregulation of the cytotoxic mediators NOX2 and iNOS (Apolloni et al., 2013a). These results are in contradiction with *in vitro* studies showing that P2X7 activation enhanced NOX2 activity in mouse primary microglia (Apolloni et al., 2013b). This discrepancy may be due in part to the origin of microglia, spinal cord *vs* brain cortex, and also to the environment of the cells. Indeed, depending on the level of activation, P2X7 can induce different polarization states of microglia. Brief activation of primary mouse microglia from SOD1-G93A mice was shown to promote autophagy while a sustained challenge increased inflammatory mediators’ expression and inhibited autophagy (Fabbrizio et al., 2017). Thus, at the first stage of the disease, P2X7 may have a beneficial role by activating autophagy while persistent stimulation of P2X7 may inhibit autophagy and contribute to pathological inflammatory responses. These results indicate that P2X7 may have a dual role in ALS by controlling autophagy and NOX2 activation (Figures 3, 4).

*In vitro*, short stimulation of P2X7 with ATP or Bz-ATP induces production of endocannabinoids by mouse microglia that may have beneficial effects (Witting et al., 2004). Indeed, the endocannabinoid, 2-arachidonoylglycerol, activates neuronal CB1 receptors which reduce glutamate release and inhibits excitotoxicity decreasing tissue destruction. Accordingly, the lack of P2X7 during experimental autoimmune encephalomyelitis (EAE), an animal model of multiple sclerosis (MS), results in the production of lower levels of endocannabinoids (Witting et al., 2006; Figures 3, 4).

Alzheimer’s disease is characterized by two main histological lesions: senile plaques composed of extracellular aggregates of amyloid β (Aβ) peptides and neurofibrillary tangles composed of intracellular aggregates of hyperphosphorylated tau protein. Using pharmacological inhibitors and neural cells from P2X7-deficient mice, we demonstrated that brief stimulation of P2X7 with ATP or Bz-ATP triggers the α-cleavage of the amyloid precursor protein (APP) (Delarasse et al., 2011; Darmellah et al., 2012). This beneficial pathway generates the neurotrophic and neuroprotective fragment sAPPα and simultaneously decreases the amount of toxic Aβ peptides. However, Leon-Otegui et al. (2011) showed, that sustained P2X7 stimulation with Bz-ATP on Neuro-2a cells activates the opposite pathway leading to Aβ peptides production. We thus explored the role of P2X7 *in vivo* in a mouse model of amyloid lesion, APPPS1 mice (Martin et al., 2019). We observed that lack of P2X7 in this model reduces the amyloid load in the brain independently of the sAPPα pathway. These studies highlight the conflicting roles attributed to P2X7 that may depend on the micro-environment in the brain. We can hypothesize that in physiological conditions, P2X7 may contribute to sAPPα release shown to trigger neurite outgrowth, synaptogenesis and proliferation of NPCs (Mattson, 1997; Caille et al., 2004). On the contrary, in the brain of AD mice, P2X7 no longer possess this property.

In the mouse amyloid model, J20, inhibition of P2X7 by the antagonist blue brilliant G was shown to reduce glycogen synthase kinase 3-beta activity, an important tau kinase (Diaz-Hernandez et al., 2012). This suggests a potential involvement of P2X7 in tau phosphorylation, however the effect of P2X7 on Tau pathology remains to be explored.

### P2X7 Roles in Inflammatory Responses

Experimental evidence suggest that P2X7 contributes to AD inflammatory processes by activating NLRP3. Indeed, NLRP3 was shown to be an important mediator of neuroinflammatory responses in AD (Halle et al., 2008; Heneka et al., 2013) and intra-hippocampal injection of Aβ in mice increases the amount of IL-1β produced, only in the presence of an active P2X7 (Rampe et al., 2004; Sanz et al., 2009). However, we showed that P2X7-deficiency in transgenic AD mice did not significantly affect the release of IL-1β in the brain at advanced and very late stages of the disease. Instead, P2X7 plays a critical role in Aβ peptide-mediated release of chemokines, particularly by increasing CCL3 levels. In effect, lower levels of CCL3 were detected in the brain of P2X7-deficient AD mice (Martin et al., 2019). In rat primary microglial cells, Kataoka et al. (2009) showed that ATP or Bz-ATP stimulation induced CCL3 release, which was partially blocked by pre-treatment with the antagonist BBG. In agreement with these results, we found no evidence of CCL3 release in the supernatants of microglia and astrocytes from P2X7-deficient mice after ATP, Bz-ATP or Aβ stimulation compared to WT cells (Martin et al., 2019). The discrepancy between acute (Sanz et al., 2009) vs. chronic AD models (Martin et al., 2019) may be explained by different activation status of microglia leading to the release of different inflammatory mediators after Aβ-stimulation of P2X7. For instance, in the absence of pre-stimulation with LPS, we observed CCL3 production in the supernatant of microglia that had been stimulated with ATP or Aβ peptides but we could not detect IL-1β release (Martin et al., 2019). Thus, we can hypothesize that, in the acute but not in the chronic AD model, the amount of ATP released, the P2X7 expression levels and the activation status of microglia favor P2X7-dependent IL-1β release (reviewed in Illes et al., 2019), but not in the chronic model. Interestingly, in mouse transgenic AD model, P2X7-dependent CCL3 release was associated with pathogenic CD8^+^ T cell recruitment (Figures 3, 4). This result supports the notion that P2X7 may be indirectly involved also in the recruitment of T lymphocytes in this disease, illustrating the complex involvement of P2X7 in inflammatory responses (Martin et al., 2019). Two different functions of P2X7, release of IL-1β and CCL3, have been highlighted in AD models and shown to contribute to the development of the disease. Thus, P2X7 antagonists are potentially pertinent pharmacological molecules to treat AD patients.

Likewise, depending on the AMD model studied, P2X7 appears to be involved in different ways. In physiological conditions, P2X7 was shown to contribute to the homeostasis of the retina as a scavenger receptor (Vessey et al., 2017). With age or light-challenge, CX3CR1-deficient mice develop subretinal macrophage accumulation associated with a significant loss of photoreceptors (Hu et al., 2015). In this model, P2X7 was up-regulated, leading to increased production of IL-1β. Treatment of CX3CR1-deficient mice with the P2X7 antagonist blue brilliant G (BBG) inhibited photoreceptor degeneration associated with subretinal inflammation (Hu et al., 2015). This study indicates that P2X7 up-regulation may have a pathogenic role in AMD via IL-1β release. Moreover, P2X7-deficiency did not affect photoreceptor loss after experimental retinal detachment, which could be explained by lower ATP and/or P2X7 levels in this model (Hu et al., 2015). There are two types of AMD, dry and wet, classified by the presence (wet) or absence (dry) of blood vessels that have disruptively invaded the retina from the choroid. In a model of dry AMD, P2X7 was shown to contribute to pathological processes via NLRP3 activation. In this model, P2X7-deficiency suppressed Alu RNA-induced RPE degeneration and caspase 1 activation (Kerur et al., 2013). In contrast, in a model of wet form of AMD, lack of P2X7 did not impact the volume of laser-induced choroidal neovascularization (CNV) but decreased the level of VEGF-A in the RPE and choroid (Mizutani et al., 2015). Interestingly, it was shown previously that stimulation of P2X7 with ATP or Bz-ATP triggers VEGF release from primary human monocyte (Hill et al., 2010) and also that VEGF secretion is reduced in P2X7-silenced B16 melanoma cells compared to WT B16 cells (Adinolfi et al., 2012). However, the cell origin of VEGF in the eye still has to be determined considering that P2X7 is exclusively expressed by microglia and endothelial cells in adult mouse retina (Kaczmarek-Hajek et al., 2018).

In summary, in AMD, P2X7 may act on different pathways such as phagocytosis, NLRP3 activation and/or VEGF-A production depending on the stage of the disease and the type of AMD (Figures 3, 4). Intrinsic defect of P2X7 could lead to the development of AMD, while overactivation of P2X7 at late stage contributes to pathological processes via release of cytokines and production of VEGF.

### P2X7 Role in Autoimmune Responses in the CNS

Multiple sclerosis is a demyelinating disease of the CNS characterized by an autoimmune response against myelin proteins. The role of P2X7 was assessed in experimental autoimmune encephalomyelitis (EAE), a mouse model of multiple sclerosis (MS). Brosnan’s group used P2X7ko mice from Pfizer and observed that P2X7-deficient mice were more susceptible to EAE (Chen and Brosnan, 2006). This effect was due to a decreased number of apoptotic T lymphocytes in the brain and spinal cord of P2X7ko vs. WT mice, indicating that P2X7 is involved in autoreactive T cell death during EAE. Similarly, a recent study reported that retinoic acid up-regulates P2X7 in effector T cells of the intestine, thereby increasing their susceptibility to P2X7-dependent apoptosis. Intestinal T-cells are in contact with the gut microbiota and the authors have shown that this enhanced P2X7 expression is instrumental in the fine regulation of T-cell populations to avoid adverse inflammatory responses (Hashimoto-Hill et al., 2017). Taken together, these two studies emphasize the role of P2X7 in the control of deleterious T-cell responses by inducing T cell apoptosis (Figures 3, 4).

**FIGURE 5:**
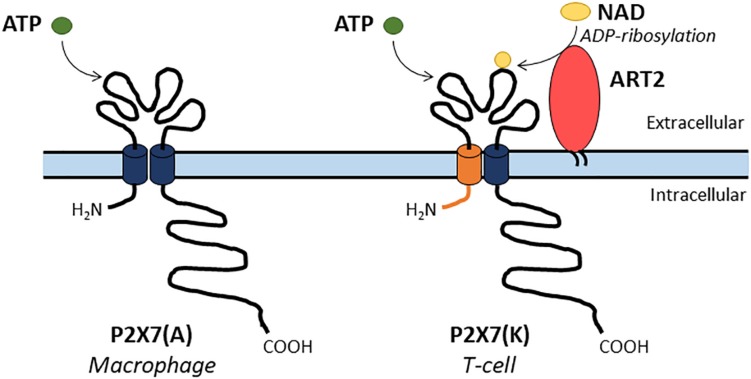
Two main P2X7 isoforms have been described in mouse. A full-length P2X7(A) isoform is expressed by macrophages while the P2X7(K) isoform is expressed by T-cells and presents an alternate NH_2_ and first transmembrane domains (depicted in orange). The P2X7(A) isoform is sensitive to ATP only while the P2X7(K) isoform is activated by both ATP or by covalent ADP-ribosylation by the enzyme ART2 in the presence of NAD.

In contrast, Sharp et al. (2008) observed that EAE incidence is reduced in P2X7-deficient mice when compared to WT animals. The discrepancy between these results and those of (Chen and Brosnan, 2006) come from the use of two different P2X7ko strains. Sharp et al. (2008) used the P2X7ko mice from Glaxo-Smith-Kline (GSK), that still express the P2X7(K) isoform, preferentially expressed in T-cells, but not the P2X7(A) isoform, present in macrophages and dendritic cells (Nicke et al., 2009; Taylor et al., 2009; Box 1 and Figure 5). Thus, in the GSK P2X7ko mice, pathogenic T cells are eliminated in part by P2X7-dependent apoptosis in the CNS during EAE as observed in WT mice.

**Box 1. Additional background information on P2X7**

• The P2X7 sensitivity varies from 10 to 100-fold between species with agonist sensitivities of human P2X7 > rat P2X7 > mouse P2X7. P2X7 sensitivities to ATP and Bz-ATP vary between species (Donnelly-Roberts et al., 2009; Bartlett et al., 2014).
• Until the development of a new generation of P2X7 antagonists, a decade ago; the antagonists of P2X7 lacked specificity. For example blue brilliant G (BBG) also inhibits P2X1R and P2X4R (Young and Gorecki, 2018).
• In murine species, two main P2X7 isoforms were described. P2X7 isoform A (P2X7(A)) is the original full-length protein and P2X7 isoform K (P2X7(K)) results from the splicing of an alternative exon 1. Thus, P2X7(A) and (K) have different amino-acid residues in their NH2 and first transmembrane domains. In addition, P2X7(K) isoform which has an 8-fold higher ATP sensitivity than P2X7A, transduces signals more efficiently (Nicke et al., 2009; Bartlett et al., 2014; Rissiek et al., 2015). P2X7(A) and (K) are preferentially expressed by macrophages or T-lymphocytes, respectively (Figure 5).
• Two main P2X7 knock-out mice were studied. The Pfizer P2X7ko was generated by inserting a neomycin cassette into exon 13 (Solle et al., 2001) while the GlaxoSmithKline one was produced by inserting a lacZ transgene and a neomycin cassette into exon 1. It was found that the GSK P2X7ko strain of mice still expresses P2X7(K) isoform in T cells (Chessell et al., 2005), reviewed in Bartlett et al. (2014).
• In mouse, nicotinamide adenine dinucleotide (NAD) can also activate P2X7 via the transfer of an ADP-ribose group from NAD to P2X7. ADP-ribosylation of P2X7 is catalyzed by the ecto-ADP-ribosyltransferase ARTC2.2. T-cells are sensitive to NAD but not macrophages, this was attributed to their differential expression of P2X7 isoforms (Seman et al., 2003; Rissiek et al., 2015; Figure 5). In human, NAD is not an agonist of P2X7 because functional ART enzyme is not present on the cell surface of lymphocytes and macrophages.

The persistence of P2X7(K)^+^ T cells in the GSK P2X7ko animals does not explain why EAE incidence is reduced in the GSK P2X7ko mice compared to WT mice. We hypothesize that the lack of P2X7(A) isoform from macrophages, dendritic cells, microglia, mast cells and astrocytes could decrease the contribution of these cells to inflammatory responses during EAE. In particular, mast cells which are considered to be important actors in EAE (Secor et al., 2000). probably lack P2X7(A) isoform in the GSK P2X7ko animals and thus will not open the brain blood barrier efficiently (Christy et al., 2013), will release lower levels of IL-1β and trigger less effectively T lymphocytes to produce GM-CSF (Russi et al., 2016). In addition, in the GSK P2X7ko mice the pathogenic macrophages have probably lost the P2X7(A) isoform and are unable to release IL-1β and other inflammatory molecules as WT macrophages do. Several reports have established that platelets contribute to pathological events during EAE (Langer et al., 2012; Starossom et al., 2015; Sonia et al., 2018). Platelets are found in the CNS before infiltration by encephalitogenic T lymphocytes (Sonia et al., 2018). They express P2Y1 and P2Y12 G-protein-coupled receptors and P2X1 only (Kahner et al., 2006; Hechler and Gachet, 2011; Mahaut-Smith et al., 2011). At early stages of EAE, platelets are able to degranulate and release several soluble factors in the CNS. Since platelets contains very large concentrations of ATP, they may activate P2X7(A) positive cells and increase inflammation in WT mice but not in GSK P2X7ko animals. P2X7 stimulation with ATP leads to CCL3 release by mouse primary microglia and astrocytes (Martin et al., 2019) and P2X7 activation with Bz-ATP induces CCL2 production by rat primary astrocytes (Panenka et al., 2001). These chemokines are involved in the recruitment of leucocytes, thus lack of P2X7 on glial cells may reduce infiltration of auto-reactive T-cells and macrophages in the brain. In addition, the disappearance of P2X7(A) isoform on oligodendrocytes could reduce their death and thus decrease demyelination (Domercq and Matute, 2019). Overall, this should lessen tissue lesions in the CNS and reduces EAE pathology in GSK P2X7ko mice.

The expression of P2X7 on HEK293 (stably transfected with P2X7) or increased expression of P2X7 in astrocyte in the brain of mice with sublethal ischemia, as well as long stimulation by Bz-ATP of primary mouse astrocyte triggers the expression of HIF-1α (Amoroso et al., 2012; Hirayama et al., 2015). HIF-1α is a metabolic sensor that decreases the number of Foxp3^+^ regulatory T cells and stimulates Th17 cell development. Notably, HIF-1α ko mice are resistant to Th17-dependent EAE induction (Dang et al., 2011). In addition, P2X7 activation also suppressed type 1 regulatory T-cell differentiation (Tr1) via an increased expression of HIF-1α in Tr1 lymphocytes (Mascanfroni et al., 2015). Thus, P2X7 might also be involved in EAE by controlling the balance between effector and regulatory T-cells via HIF-1α expression.

Genome-wide associated studies (GWAS) of patients with MS (International Multiple Sclerosis Genetics Consortium, 2007; Australia and New Zealand Multiple Sclerosis Genetics Consortium, 2009; International Multiple Sclerosis Genetics Consortium, 2011, 2013, 2018) identified multiple gene alleles associated with higher risk of MS but none of the highly polymorphic P2X7 genes. The frequency of P2X7 variants may vary depending on the variants studied (rare: mean allele frequency (MAF) <3% or common: MAF > 5%) and on the localization of the populations considered, i.e., a given P2X7 variant may be rare in certain groups and absent in others. When the frequency of the variants is rare, this low frequency may not be sufficient to determine whether certain variants are associated with particular pathologies through the GWAS method.

Gu et al. (2015) have genotyped 12 functional P2X7 variants in three case-control cohorts of MS patients and normal subjects. Interestingly, they identified a rare genetic variant of P2X7 R307Q that has lost P2X7 pore function and shows a protective effect against MS. In addition, they found that the R307Q variant is associated with another variant R270H with a partial loss of P2X7 pore function. In patients, the combination of these two variants may impair P2X7 function and reduces secretion of pro-inflammatory cytokines by activated macrophages in the CNS (Gu et al., 2015; Sadovnick et al., 2017). Sadovnick et al. (2017) sequenced the P2X7 and P2X4 of MS patients and healthy controls. They identified three rare variants (P2X7 T205M, P2X7 N361 S and P2X4 G135S) which co-segregate with MS in one family in which 6 individuals out of 13 were affected by the disease. The P2X7 T205M mutant has lost its plasma membrane expression and its phagocytic capacity. The two other mutations, P2X7 N361 S and P2X4 G135S, had minor impact on P2X4-P2X7 functions but additional studies are needed to determine whether these two rare variants are also risk factors in MS. These studies have shown that the mutations affect P2X7 functions in macrophages, however, it would be of great interest to evaluate whether these mutations affect T-lymphocytes which play a major role in MS.

To identify risk loci in late onset AD, several GWAS of AD patients were performed (Harold et al., 2009; Lambert et al., 2009; Jun et al., 2010; Hollingworth et al., 2011; Naj et al., 2011; Cruchaga et al., 2013; Lambert et al., 2013; Ramanan and Saykin, 2013; Marioni et al., 2018; Jansen et al., 2019; Kunkle et al., 2019). In these studies, about 29 loci were identified to be associated with higher risk of developing AD, but they did not include variants of *P2*X*7* gene. However, disease susceptibility may be due to the combined roles of different gene polymorphisms, which have minor individual effects but synergize when associated. Analyses of several linked variants could reveal the involvement of a particular biological pathway in the disease process (Marchini et al., 2005; Damotte et al., 2014). Thus, we performed gene pathway analyses based on published GWAS data (Lambert et al., 2009; Lambert et al., 2013). We analyzed polymorphisms in the *P2*X*7* gene as well as those found in genes involved in P2X7-dependent sAPPα release, NALP3/ASC inflammasome and caspase 1 activation, IL-1β production and IL-1β receptor genes (IL-1R1, IL-1R2, and IL-1RAcP). A global analysis of P2X7 pathways and a more detailed study of subnetworks did not identify any association with AD (unpublished observations). We believe that the strategy used by Gu et al. (2015) to identify P2X7 rare variants associated with MS should be applied to AD.

## Conclusion

This review highlights the difficulties encountered in assigning a specific role to P2X7 in the nervous system (Figures 3, 4). Whether beneficial or detrimental, the effects of P2X7 in neurological diseases vary, depending on the disease physiopathology and clinical stage. The impact of P2X7 in the CNS is determined by numerous factors such as (1) cell types expressing it, (2) levels of P2X7 at the cell surface, (3) isoforms of P2X7 expressed, (4) biochemical pathways triggered in different cell types, (5) the local concentrations of ATP and (6) the activities of ecto-nucleotidases. The complexity of P2X7 functions is underscored by the variety of responses triggered by P2X7 stimulation on the same cell type. Indeed, P2X7 stimulation of microglia leads to CCL3 secretion while IL-1β processing and release requires pre-stimulation by TLR ligands. In addition, several cytokines such as INFγ and TNFα trigger P2X7 expression in various cell types. Other factors that influence the lipid composition of plasma membrane could modify the localization of P2X7 in or out the lipid rafts on the cell surface. Consequently, this affects the formation of the non-selective pore and the stimulation of intracellular signaling pathways (Garcia-Marcos et al., 2006; Karasawa and Kawate, 2016). The level of ATP also influences P2X7 properties. Differentiation or cell death depend on ATP concentrations, as was shown for adult NPC (Leeson et al., 2018). In the absence of ATP and serum, it was clearly shown that P2X7 confers scavenger activity to monocytes and microglia (Gu et al., 2011; Gu and Wiley, 2018; Janks et al., 2018). However, at a later stage of the disease, when higher amounts of ATP are released, P2X7 has a pro-inflammatory role. In addition, several ecto-nucleotidases expressed at the cell surface may degrade ATP in ADP, AMP and adenosine and thus activate other purinergic receptors (Rodrigues et al., 2015). These receptors contribute to ATP release and can synergize or antagonize P2X7 functions. For example the expression of P2Y1 that induces the proliferation of NPCs may counterbalance P2X7-dependent cell death (Rozmer et al., 2017). P2X4 activation leads to BDNF production and release supporting remyelination after P2X7-mediated myelin damage (Domercq and Matute, 2019). In contrast, inhibition of P2X7 or of the adenosine receptor A2A is beneficial in mouse amyloid models of AD (Faivre et al., 2018; Martin et al., 2019). Data on *in vivo* physiological levels of ATP in the CNS and during neurodegenerative diseases would be a major information to determine the relevant functions of P2X7. Luciferase probes were developed to measure ATP in animal models of colitis or tumor (Morciano et al., 2017), it would be very interesting to optimize this protocol for CNS related diseases.

The complex role of P2X7 in CNS diseases is reminiscent of the dual role of the Fas receptor and its ligand (FasL) in the pathogenesis and evolution of EAE (Sabelko-Downes et al., 1999). In their review, Sabelko-Downes et al. (1999) proposed a model in which encephalitogenic FasL^+^ CD4^+^ T lymphocytes enter the CNS and induce apoptosis of Fas^+^ resident cells such as oligodendrocytes. Importantly, during the recovery phase of EAE, encephalitogenic T lymphocytes express Fas while the cytokine-stimulated CNS resident cells become FasL^+^. Thus, they can eliminate the aggressive CD4^+^ T cells by binding the Fas receptor. It is worth noticing that in EAE, P2X7 present on encephalitogenic T lymphocytes contributes to the elimination of T lymphocytes during the remission phase of the disease (Chen and Brosnan, 2006). In all these studies, the use of Fas/FasL or P2X7 deficient mice was of major interest in delineating the role of these receptors in EAE. However, the removal of P2X7 from a large number of cell types expressing it precludes refined analyses of its functions on well-characterized cell sub-populations. The availability of P2X7 conditional ko mice (Csoka et al., 2015) in which it is possible to restrain and/or induce the elimination of this receptor in CNS cell subpopulations would be of major interest to define its role in physiological or pathological situations.

P2X7 as a potential therapeutic target has been investigated in numerous diseases such as chronic inflammatory diseases, neurodegenerative pathologies associated with inflammation, cancer and mental disorders (reviewed in Di Virgilio et al., 2017; Savio et al., 2018; Young and Gorecki, 2018). Several pharmaceutical companies have produced pharmacological antagonists of P2X7 which have been tested in various diseases with mostly disappointing results (Di Virgilio et al., 2017; Savio et al., 2018; Young and Gorecki, 2018). However, recently, instead of using small molecules able to antagonize P2X7, Danquah et al. (2016) have immunized llamas with mouse or human P2X7 in native conformation and selected nanobodies able to inhibit or trigger P2X7 functions. They were able to identify one potent agonist of mouse P2X7, nanobody 14D5 and one potent antagonist, nanobody 13A7. They showed that 13A7 bivalent nanobodies were able to block P2X7 functions on primary mouse macrophages and T lymphocytes and significantly decreased inflammation in two mouse models: allergic contact dermatitis and experimental glomerulonephritis (Danquah et al., 2016). The potential use of these anti-P2X7 nanobodies with antagonist properties in human inflammation is strengthened by experiments showing that the anti-human P2X7 antagonist nanobody Dano1 inhibits IL-1β release from human monocytes at subnanomolar IC50. Interestingly, the inhibitory potency of Dano1 was 1000 times higher than two pharmacological inhibitors of P2X7 used in clinical trials (Danquah et al., 2016). These new nanobodies with powerful antagonist properties against the mouse and human P2X7 show promising therapeutic potential in various inflammatory and neurologic diseases (reviewed in Koch-Nolte et al., 2019).

## Author Contributions

JK and CD wrote the manuscript.

## Conflict of Interest Statement

The authors declare that the research was conducted in the absence of any commercial or financial relationships that could be construed as a potential conflict of interest.

## References

[B1] Adinolfi E. Callegari M. G. Cirillo M. Pinton P. Giorgi C. Cavagna D. (2009). Expression of the P2X7 receptor increases the Ca2+ content of the endoplasmic reticulum, activates NFATc1, and protects from apoptosis. *J. Biol. Chem.* 284 10120–10128. 10.1074/jbc.M805805200 19204004 PMC2665066

[B2] Adinolfi E. Callegari M. G. Ferrari D. Bolognesi C. Minelli M. Wieckowski M. R. (2005). Basal activation of the P2X7 ATP receptor elevates mitochondrial calcium and potential, increases cellular ATP levels, and promotes serum-independent growth. *Mol. Biol. Cell* 16 3260–3272. 10.1091/mbc.e04-11-1025 15901833 PMC1165409

[B3] Adinolfi E. Cirillo M. Woltersdorf R. Falzoni S. Chiozzi P. Pellegatti P. (2010). Trophic activity of a naturally occurring truncated isoform of the P2X7 receptor. *FASEB J.* 24 3393–3404. 10.1096/fj.09-153601 20453110

[B4] Adinolfi E. Raffaghello L. Giuliani A. L. Cavazzini L. Capece M. Chiozzi P. (2012). Expression of P2X7 receptor increases in vivo tumor growth. *Cancer Res.* 72 2957–2969. 10.1158/0008-5472.CAN-11-1947 22505653

[B5] Aglietti R. A. Estevez A. Gupta A. Ramirez M. G. Liu P. S. Kayagaki N. (2016). GsdmD p30 elicited by caspase-11 during pyroptosis forms pores in membranes. *Proc. Natl. Acad. Sci. U.S.A.* 113 7858–7863. 10.1073/pnas.1607769113 27339137 PMC4948338

[B6] Alberto A. V. Faria R. X. Couto C. G. Ferreira L. G. Souza C. A. Teixeira P. C. (2013). Is pannexin the pore associated with the P2X7 receptor? *Naunyn Schmiedebergs Arch. Pharmacol.* 386 775–787. 10.1007/s00210-013-0868-x 23657251

[B7] Amadio S. Parisi C. Piras E. Fabbrizio P. Apolloni S. Montilli C. (2017). Modulation of P2X7 receptor during inflammation in multiple sclerosis. *Front. Immunol.* 8:1529. 10.3389/fimmu.2017.01529 29187851 PMC5694754

[B8] Amoroso F. Falzoni S. Adinolfi E. Ferrari D. Di Virgilio F. (2012). The P2X7 receptor is a key modulator of aerobic glycolysis. *Cell Death Dis.* 3:e370. 10.1038/cddis.2012.105 22898868 PMC3434661

[B9] Andrei C. Dazzi C. Lotti L. Torrisi M. R. Chimini G. Rubartelli A. (1999). The secretory route of the leaderless protein interleukin 1beta involves exocytosis of endolysosome-related vesicles. *Mol. Biol. Cell* 10 1463–1475. 10.1091/mbc.10.5.1463 10233156 PMC25302

[B10] Andrei C. Margiocco P. Poggi A. Lotti L. V. Torrisi M. R. Rubartelli A. (2004). Phospholipases C and A2 control lysosome-mediated IL-1 beta secretion: implications for inflammatory processes. *Proc. Natl. Acad. Sci. U.S.A.* 101 9745–9750. 10.1073/pnas.0308558101 15192144 PMC470745

[B11] Apolloni S. Amadio S. Montilli C. Volonte C. D’ambrosi N. (2013a). Ablation of P2X7 receptor exacerbates gliosis and motoneuron death in the SOD1-G93A mouse model of amyotrophic lateral sclerosis. *Hum. Mol. Genet.* 22 4102–4116. 10.1093/hmg/ddt259 23736299

[B12] Apolloni S. Parisi C. Pesaresi M. G. Rossi S. Carri M. T. Cozzolino M. (2013b). The NADPH oxidase pathway is dysregulated by the P2X7 receptor in the SOD1-G93A microglia model of amyotrophic lateral sclerosis. *J. Immunol.* 190 5187–5195. 10.4049/jimmunol.1203262 23589615

[B13] Auger R. Motta I. Benihoud K. Ojcius D. M. Kanellopoulos J. M. (2005). A role for mitogen-activated protein kinase(Erk1/2) activation and non-selective pore formation in P2X7 receptor-mediated thymocyte death. *J. Biol. Chem.* 280 28142–28151. 10.1074/jbc.m501290200 15937334

[B14] Australia and New Zealand Multiple Sclerosis Genetics Consortium (2009). Genome-wide association study identifies new multiple sclerosis susceptibility loci on chromosomes 12 and 20. *Nat. Genet.* 41 824–828. 10.1038/ng.396 19525955

[B15] Baricordi O. R. Melchiorri L. Adinolfi E. Falzoni S. Chiozzi P. Buell G. (1999). Increased proliferation rate of lymphoid cells transfected with the P2X(7) ATP receptor. *J. Biol. Chem.* 274 33206–33208. 10.1074/jbc.274.47.33206 10559192

[B16] Barros-Barbosa A. R. Fonseca A. L. Guerra-Gomes S. Ferreirinha F. Santos A. Rangel R. (2016). Up-regulation of P2X7 receptor-mediated inhibition of GABA uptake by nerve terminals of the human epileptic neocortex. *Epilepsia* 57 99–110. 10.1111/epi.13263 26714441

[B17] Barth K. Weinhold K. Guenther A. Young M. T. Schnittler H. Kasper M. (2007). Caveolin-1 influences P2X7 receptor expression and localization in mouse lung alveolar epithelial cells. *FEBS J.* 274 3021–3033. 10.1111/j.1742-4658.2007.05830.x 17498208

[B18] Bartlett R. Stokes L. Sluyter R. (2014). The P2X7 receptor channel: recent developments and the use of P2X7 antagonists in models of disease. *Pharmacol. Rev.* 66 638–675. 10.1124/pr.113.008003 24928329

[B19] Beamer E. Fischer W. Engel T. (2017). The ATP-Gated P2X7 receptor as a target for the treatment of drug-resistant epilepsy. *Front. Neurosci.* 11:21. 10.3389/fnins.2017.00021 28210205 PMC5288361

[B20] Beyer E. C. Steinberg T. H. (1991). Evidence that the gap junction protein connexin-43 is the ATP-induced pore of mouse macrophages. *J. Biol. Chem.* 266 7971–7974. 1708769

[B21] Bianco F. Ceruti S. Colombo A. Fumagalli M. Ferrari D. Pizzirani C. (2006). A role for P2X7 in microglial proliferation. *J. Neurochem.* 99 745–758. 16836656 10.1111/j.1471-4159.2006.04101.x

[B22] Burnstock G. (1972). Purinergic nerves. *Pharmacol. Rev.* 24 509–581. 4404211

[B23] Burnstock G. Satchell D. G. Smythe A. (1972). A comparison of the excitatory and inhibitory effects of non-adrenergic, non-cholinergic nerve stimulation and exogenously applied ATP on a variety of smooth muscle preparations from different vertebrate species. *Br. J. Pharmacol.* 46 234–242. 10.1111/j.1476-5381.1972.tb06868.x 4631338 PMC1666337

[B24] Caille I. Allinquant B. Dupont E. Bouillot C. Langer A. Muller U. (2004). Soluble form of amyloid precursor protein regulates proliferation of progenitors in the adult subventricular zone. *Development* 131 2173–2181. 10.1242/dev.01103 15073156

[B25] Chen G. Y. Nunez G. (2010). Sterile inflammation: sensing and reacting to damage. *Nat. Rev. Immunol.* 10 826–837. 10.1038/nri2873 21088683 PMC3114424

[B26] Chen L. Brosnan C. F. (2006). Exacerbation of experimental autoimmune encephalomyelitis in P2X7R-/- mice: evidence for loss of apoptotic activity in lymphocytes. *J. Immunol.* 176 3115–3126. 10.4049/jimmunol.176.5.3115 16493071

[B27] Chessell I. P. Hatcher J. P. Bountra C. Michel A. D. Hughes J. P. Green P. (2005). Disruption of the P2X7 purinoceptor gene abolishes chronic inflammatory and neuropathic pain. *Pain* 114 386–396. 10.1016/j.pain.2005.01.002 15777864

[B28] Christy A. L. Walker M. E. Hessner M. J. Brown M. A. (2013). Mast cell activation and neutrophil recruitment promotes early and robust inflammation in the meninges in EAE. *J. Autoimmun.* 42 50–61. 10.1016/j.jaut.2012.11.003 23267561

[B29] Cruchaga C. Kauwe J. S. Harari O. Jin S. C. Cai Y. Karch C. M. (2013). GWAS of cerebrospinal fluid tau levels identifies risk variants for Alzheimer’s disease. *Neuron* 78 256–268. 10.1016/j.neuron.2013.02.026 23562540 PMC3664945

[B30] Csoka B. Nemeth Z. H. Toro G. Idzko M. Zech A. Koscso B. (2015). Extracellular ATP protects against sepsis through macrophage P2X7 purinergic receptors by enhancing intracellular bacterial killing. *FASEB J.* 29 3626–3637. 10.1096/fj.15-272450 26060214 PMC4550379

[B31] Cullen S. P. Kearney C. J. Clancy D. M. Martin S. J. (2015). Diverse Activators of the NLRP3 inflammasome promote IL-1beta secretion by triggering necrosis. *Cell Rep.* 11 1535–1548. 10.1016/j.celrep.2015.05.003 26027935

[B32] Damotte V. Guillot-Noel L. Patsopoulos N. A. Madireddy L. El Behi M. International Multiple Sclerosis Genetics Consortium (2014). A gene pathway analysis highlights the role of cellular adhesion molecules in multiple sclerosis susceptibility. *Genes Immun.* 15 126–132. 10.1038/gene.2013.70 24430173

[B33] Dang E. V. Barbi J. Yang H. Y. Jinasena D. Yu H. Zheng Y. (2011). Control of T(H)17/T(reg) balance by hypoxia-inducible factor 1. *Cell* 146 772–784. 10.1016/j.cell.2011.07.033 21871655 PMC3387678

[B34] Danquah W. Meyer-Schwesinger C. Rissiek B. Pinto C. Serracant-Prat A. Amadi M. (2016). Nanobodies that block gating of the P2X7 ion channel ameliorate inflammation. *Sci. Transl. Med.* 8:366ra162. 10.1126/scitranslmed.aaf8463 27881823

[B35] Darmellah A. Rayah A. Auger R. Cuif M. H. Prigent M. Arpin M. (2012). Ezrin/radixin/moesin are required for the purinergic P2X7 receptor (P2X7R)-dependent processing of the amyloid precursor protein. *J. Biol. Chem.* 287 34583–34595. 10.1074/jbc.M112.400010 22891241 PMC3464564

[B36] Delarasse C. Auger R. Gonnord P. Fontaine B. Kanellopoulos J. M. (2011). The purinergic receptor P2X7 triggers alpha-secretase-dependent processing of the amyloid precursor protein. *J. Biol. Chem.* 286 2596–2606. 10.1074/jbc.M110.200618 21081501 PMC3024755

[B37] Delarasse C. Gonnord P. Galante M. Auger R. Daniel H. Motta I. (2009). Neural progenitor cell death is induced by extracellular ATP via ligation of P2X7 receptor. *J. Neurochem.* 109 846–857. 10.1111/j.1471-4159.2009.06008.x 19250337

[B38] Di A. Xiong S. Ye Z. Malireddi R. K. S. Kometani S. Zhong M. (2018). The TWIK2 potassium efflux channel in macrophages mediates NLRP3 inflammasome-induced inflammation. *Immunity* 49 56–65.e4. 10.1016/j.immuni.2018.04.032 29958799 PMC6051907

[B39] Di Virgilio F. (2007). Liaisons dangereuses: P2X(7) and the inflammasome. *Trends Pharmacol. Sci.* 28 465–472. 10.1016/j.tips.2007.07.002 17692395

[B40] Di Virgilio F. Dal Ben D. Sarti A. C. Giuliani A. L. Falzoni S. (2017). The P2X7 receptor in infection and inflammation. *Immunity* 47 15–31. 10.1016/j.immuni.2017.06.020 28723547

[B41] Di Virgilio F. Schmalzing G. Markwardt F. (2018). The elusive P2X7 macropore. *Trends Cell Biol.* 28 392–404. 10.1016/j.tcb.2018.01.005 29439897

[B42] Diaz-Hernandez J. I. Gomez-Villafuertes R. Leon-Otegui M. Hontecillas-Prieto L. Del Puerto A. Trejo J. L. (2012). In vivo P2X7 inhibition reduces amyloid plaques in Alzheimer’s disease through GSK3beta and secretases. *Neurobiol. Aging* 33 1816–1828. 10.1016/j.neurobiolaging.2011.09.040 22048123

[B43] Ding J. Wang K. Liu W. She Y. Sun Q. Shi J. (2016). Pore-forming activity and structural autoinhibition of the gasdermin family. *Nature* 535 111–116. 10.1038/nature18590 27281216

[B44] Domercq M. Matute C. (2019). Targeting P2X4 and P2X7 receptors in multiple sclerosis. *Curr. Opin. Pharmacol.* 47 119–125. 10.1016/j.coph.2019.03.010 31015145

[B45] Domingo-Fernandez R. Coll R. C. Kearney J. Breit S. O’Neill L. A. J. (2017). The intracellular chloride channel proteins CLIC1 and CLIC4 induce IL-1beta transcription and activate the NLRP3 inflammasome. *J. Biol. Chem.* 292 12077–12087. 10.1074/jbc.M117.797126 28576828 PMC5519359

[B46] Donnelly-Roberts D. L. Namovic M. T. Faltynek C. R. Jarvis M. F. (2004). Mitogen-activated protein kinase and caspase signaling pathways are required for P2X7 receptor (P2X7R)-induced pore formation in human THP-1 cells. *J. Pharmacol. Exp. Ther.* 308 1053–1061. 10.1124/jpet.103.059600 14634045

[B47] Donnelly-Roberts D. L. Namovic M. T. Han P. Jarvis M. F. (2009). Mammalian P2X7 receptor pharmacology: comparison of recombinant mouse, rat and human P2X7 receptors. *Br. J. Pharmacol.* 157 1203–1214. 10.1111/j.1476-5381.2009.00233.x 19558545 PMC2743839

[B48] Engel T. Gomez-Villafuertes R. Tanaka K. Mesuret G. Sanz-Rodriguez A. Garcia-Huerta P. (2012). Seizure suppression and neuroprotection by targeting the purinergic P2X7 receptor during status epilepticus in mice. *FASEB J.* 26 1616–1628. 10.1096/fj.11-196089 22198387

[B49] Evavold C. L. Kagan J. C. (2018). How inflammasomes inform adaptive immunity. *J. Mol. Biol.* 430 217–237. 10.1016/j.jmb.2017.09.019 28987733 PMC5766381

[B50] Evavold C. L. Ruan J. Tan Y. Xia S. Wu H. Kagan J. C. (2018). The Pore-forming protein gasdermin D regulates interleukin-1 secretion from living macrophages. *Immunity* 48 35–44.e6. 10.1016/j.immuni.2017.11.013 29195811 PMC5773350

[B51] Fabbrizio P. Amadio S. Apolloni S. Volonte C. (2017). P2X7 receptor activation modulates autophagy in SOD1-G93A mouse microglia. *Front. Cell. Neurosci.* 11:249. 10.3389/fncel.2017.00249 28871219 PMC5566572

[B52] Faivre E. Coelho J. E. Zornbach K. Malik E. Baqi Y. Schneider M. (2018). Beneficial effect of a selective adenosine A2A receptor antagonist in the APPswe/PS1dE9 mouse model of Alzheimer’s disease. *Front. Mol. Neurosci.* 11:235. 10.3389/fnmol.2018.00235 30050407 PMC6052540

[B53] Faria R. X. Defarias F. P. Alves L. A. (2005). Are second messengers crucial for opening the pore associated with P2X7 receptor? *Am. J. Physiol. Cell Physiol.* 288 C260–C271. 15469955 10.1152/ajpcell.00215.2004

[B54] Ferrari D. Chiozzi P. Falzoni S. Dal Susino M. Melchiorri L. Baricordi O. R. (1997a). Extracellular ATP triggers IL-1 beta release by activating the purinergic P2Z receptor of human macrophages. *J. Immunol.* 159 1451–1458. 9233643

[B55] Ferrari D. Chiozzi P. Falzoni S. Hanau S. Di Virgilio F. (1997b). Purinergic modulation of interleukin-1 beta release from microglial cells stimulated with bacterial endotoxin. *J. Exp. Med.* 185 579–582. 10.1084/jem.185.3.579 9053458 PMC2196027

[B56] Garcia-Marcos M. Perez-Andres E. Tandel S. Fontanils U. Kumps A. Kabre E. (2006). Coupling of two pools of P2X7 receptors to distinct intracellular signaling pathways in rat submandibular gland. *J. Lipid Res.* 47 705–714. 10.1194/jlr.m500408-jlr200 16415476

[B57] Gonnord P. Delarasse C. Auger R. Benihoud K. Prigent M. Cuif M. H. (2009). Palmitoylation of the P2X7 receptor, an ATP-gated channel, controls its expression and association with lipid rafts. *FASEB J.* 23 795–805. 10.1096/fj.08-114637 18971257

[B58] Green J. P. Yu S. Martin-Sanchez F. Pelegrin P. Lopez-Castejon G. Lawrence C. B. (2018). Chloride regulates dynamic NLRP3-dependent ASC oligomerization and inflammasome priming. *Proc. Natl. Acad. Sci. U.S.A.* 115 E9371–E9380. 10.1073/pnas.1812744115 30232264 PMC6176575

[B59] Gu B. J. Baird P. N. Vessey K. A. Skarratt K. K. Fletcher E. L. Fuller S. J. (2013). A rare functional haplotype of the P2RX4 and P2RX7 genes leads to loss of innate phagocytosis and confers increased risk of age-related macular degeneration. *FASEB J.* 27 1479–1487. 10.1096/fj.12-215368 23303206

[B60] Gu B. J. Field J. Dutertre S. Ou A. Kilpatrick T. J. Lechner-Scott J. (2015). A rare P2X7 variant Arg307Gln with absent pore formation function protects against neuroinflammation in multiple sclerosis. *Hum. Mol. Genet.* 24 5644–5654. 10.1093/hmg/ddv278 26188005

[B61] Gu B. J. Saunders B. M. Petrou S. Wiley J. S. (2011). P2X(7) Is a scavenger receptor for apoptotic cells in the absence of its ligand, extracellular ATP. *J. Immunol.* 187 2365–2375. 10.4049/jimmunol.1101178 21821797

[B62] Gu B. J. Wiley J. S. (2018). P2X7 as a scavenger receptor for innate phagocytosis in the brain. *Br. J. Pharmacol.* 175 4195–4208. 10.1111/bph.14470 30098011 PMC6193880

[B63] Guillonneau X. Eandi C. M. Paques M. Sahel J. A. Sapieha P. Sennlaub F. (2017). On phagocytes and macular degeneration. *Prog. Retin. Eye Res.* 61 98–128. 10.1016/j.preteyeres.2017.06.002 28602950

[B64] Habbas S. Ango F. Daniel H. Galante M. (2011). Purinergic signaling in the cerebellum: bergmann glial cells express functional ionotropic P2X7 receptors. *Glia* 59 1800–1812. 10.1002/glia.21224 21830236

[B65] Halle A. Hornung V. Petzold G. C. Stewart C. R. Monks B. G. Reinheckel T. (2008). The NALP3 inflammasome is involved in the innate immune response to amyloid-beta. *Nat. Immunol.* 9 857–865. 10.1038/ni.1636 18604209 PMC3101478

[B66] Harkat M. Peverini L. Cerdan A. H. Dunning K. Beudez J. Martz A. (2017). On the permeation of large organic cations through the pore of ATP-gated P2X receptors. *Proc. Natl. Acad. Sci. U.S.A.* 114 E3786–E3795. 10.1073/pnas.1701379114 28442564 PMC5441707

[B67] Harold D. Abraham R. Hollingworth P. Sims R. Gerrish A. Hamshere M. L. (2009). Genome-wide association study identifies variants at CLU and PICALM associated with Alzheimer’s disease. *Nat. Genet.* 41 1088–1093. 10.1038/ng.440 19734902 PMC2845877

[B68] Hashimoto-Hill S. Friesen L. Kim M. Kim C. H. (2017). Contraction of intestinal effector T cells by retinoic acid-induced purinergic receptor P2X7. *Mucosal Immunol.* 10 912–923. 10.1038/mi.2016.109 27966552 PMC5471139

[B69] Hattori M. Gouaux E. (2012). Molecular mechanism of ATP binding and ion channel activation in P2X receptors. *Nature* 485 207–212. 10.1038/nature11010 22535247 PMC3391165

[B70] He Y. Taylor N. Fourgeaud L. Bhattacharya A. (2017). The role of microglial P2X7: modulation of cell death and cytokine release. *J. Neuroinflammation* 14:135. 10.1186/s12974-017-0904-8 28716092 PMC5513370

[B71] He Y. Zeng M. Y. Yang D. Motro B. Nunez G. (2016). NEK7 is an essential mediator of NLRP3 activation downstream of potassium efflux. *Nature* 530 354–357. 10.1038/nature16959 26814970 PMC4810788

[B72] Hechler B. Gachet C. (2011). P2 receptors and platelet function. *Purinergic Signal.* 7 293–303. 10.1007/s11302-011-9247-6 21792575 PMC3166986

[B73] Heilig R. Dick M. S. Sborgi L. Meunier E. Hiller S. Broz P. (2018). The Gasdermin-D pore acts as a conduit for IL-1beta secretion in mice. *Eur. J. Immunol.* 48 584–592. 10.1002/eji.201747404 29274245

[B74] Heneka M. T. Kummer M. P. Stutz A. Delekate A. Schwartz S. Vieira-Saecker A. (2013). NLRP3 is activated in Alzheimer’s disease and contributes to pathology in APP/PS1 mice. *Nature* 493 674–678. 10.1038/nature11729 23254930 PMC3812809

[B75] Hill L. M. Gavala M. L. Lenertz L. Y. Bertics P. J. (2010). Extracellular ATP may contribute to tissue repair by rapidly stimulating purinergic receptor X7-dependent vascular endothelial growth factor release from primary human monocytes. *J. Immunol.* 185 3028–3034. 10.4049/jimmunol.1001298 20668222 PMC3156583

[B76] Hirayama Y. Ikeda-Matsuo Y. Notomi S. Enaida H. Kinouchi H. Koizumi S. (2015). Astrocyte-mediated ischemic tolerance. *J. Neurosci.* 35 3794–3805. 10.1523/JNEUROSCI.4218-14.2015 25740510 PMC6605570

[B77] Hollingworth P. Harold D. Sims R. Gerrish A. Lambert J. C. Carrasquillo M. M. (2011). Common variants at ABCA7, MS4A6A/MS4A4E, EPHA1, CD33 and CD2AP are associated with Alzheimer’s disease. *Nat. Genet.* 43 429–435. 10.1038/ng.803 21460840 PMC3084173

[B78] Hu S. J. Calippe B. Lavalette S. Roubeix C. Montassar F. Housset M. (2015). Upregulation of P2RX7 in Cx3cr1-deficient mononuclear phagocytes leads to increased interleukin-1beta secretion and photoreceptor neurodegeneration. *J. Neurosci.* 35 6987–6996. 10.1523/JNEUROSCI.3955-14.2015 25948251 PMC6605270

[B79] Illes P. Khan T. M. Rubini P. (2017). Neuronal P2X7 receptors revisited: do they really exist? *J. Neurosci.* 37 7049–7062. 10.1523/JNEUROSCI.3103-16.2017 28747388 PMC6705732

[B80] Illes P. Rubini P. Huang L. Tang Y. (2019). The P2X7 receptor: a new therapeutic target in Alzheimer’s disease. *Expert. Opin. Ther. Targets* 23 165–176. 10.1080/14728222.2019.1575811 30691318

[B81] International Multiple Sclerosis Genetics Consortium (2007). Risk alleles for multiple sclerosis identified by a genomewide study. *N. Engl. J. Med.* 357 851–862. 10.1056/nejmoa073493 17660530

[B82] International Multiple Sclerosis Genetics Consortium (2011). Genetic risk and a primary role for cell-mediated immune mechanisms in multiple sclerosis. *Nature* 476 214–219. 10.1038/nature10251 21833088 PMC3182531

[B83] International Multiple Sclerosis Genetics Consortium (2013). Analysis of immune-related loci identifies 48 new susceptibility variants for multiple sclerosis. *Nat. Genet.* 45 1353–1360. 10.1038/ng.2770 24076602 PMC3832895

[B84] International Multiple Sclerosis Genetics Consortium, (2018). Low-frequency and rare-coding variation contributes to multiple sclerosis risk. *Cell* 175 1679–1687.e7. 10.1016/j.cell.2018.09.049 30343897 PMC6269166

[B85] Janks L. Sharma C. V. R. Egan T. M. (2018). A central role for P2X7 receptors in human microglia. *J. Neuroinflammation* 15:325. 10.1186/s12974-018-1353-8 30463629 PMC6247771

[B86] Jansen I. E. Savage J. E. Watanabe K. Bryois J. Williams D. M. Steinberg S. (2019). Genome-wide meta-analysis identifies new loci and functional pathways influencing Alzheimer’s disease risk. *Nat. Genet.* 51 404–413. 10.1038/s41588-018-0311-9 30617256 PMC6836675

[B87] Jimenez-Pacheco A. Diaz-Hernandez M. Arribas-Blazquez M. Sanz-Rodriguez A. Olivos-Ore L. A. Artalejo A. R. (2016). Transient P2X7 receptor antagonism produces lasting reductions in spontaneous seizures and gliosis in experimental temporal lobe epilepsy. *J. Neurosci.* 36 5920–5932. 10.1523/JNEUROSCI.4009-15.2016 27251615 PMC6601816

[B88] Jun G. Naj A. C. Beecham G. W. Wang L. S. Buros J. Gallins P. J. (2010). Meta-analysis confirms CR1, CLU, and PICALM as alzheimer disease risk loci and reveals interactions with APOE genotypes. *Arch. Neurol.* 67 1473–1484. 10.1001/archneurol.2010.201 20697030 PMC3048805

[B89] Kaczmarek-Hajek K. Zhang J. Kopp R. Grosche A. Rissiek B. Saul A. (2018). Re-evaluation of neuronal P2X7 expression using novel mouse models and a P2X7-specific nanobody. *eLife* 7:e36217. 10.7554/eLife.36217 30074479 PMC6140716

[B90] Kahner B. N. Shankar H. Murugappan S. Prasad G. L. Kunapuli S. P. (2006). Nucleotide receptor signaling in platelets. *J. Thromb. Haemost.* 4 2317–2326. 10.1111/j.1538-7836.2006.02192.x 17059469

[B91] Karasawa A. Kawate T. (2016). Structural basis for subtype-specific inhibition of the P2X7 receptor. *eLife* 5:e22153. 10.7554/eLife.22153 27935479 PMC5176352

[B92] Karasawa A. Michalski K. Mikhelzon P. Kawate T. (2017). The P2X7 receptor forms a dye-permeable pore independent of its intracellular domain but dependent on membrane lipid composition. *eLife* 6:e31186. 10.7554/eLife.31186 28920575 PMC5624784

[B93] Kasuya G. Yamaura T. Ma X. B. Nakamura R. Takemoto M. Nagumo H. (2017). Structural insights into the competitive inhibition of the ATP-gated P2X receptor channel. *Nat. Commun.* 8:876. 10.1038/s41467-017-00887-9 29026074 PMC5638823

[B94] Kataoka A. Tozaki-Saitoh H. Koga Y. Tsuda M. Inoue K. (2009). Activation of P2X7 receptors induces CCL3 production in microglial cells through transcription factor NFAT. *J. Neurochem.* 108 115–125. 10.1111/j.1471-4159.2008.05744.x 19014371

[B95] Kawate T. Michel J. C. Birdsong W. T. Gouaux E. (2009). Crystal structure of the ATP-gated P2X(4) ion channel in the closed state. *Nature* 460 592–598. 10.1038/nature08198 19641588 PMC2720809

[B96] Kayagaki N. Stowe I. B. Lee B. L. O’rourke K. Anderson K. Warming S. (2015). Caspase-11 cleaves gasdermin D for non-canonical inflammasome signalling. *Nature* 526 666–671. 10.1038/nature15541 26375259

[B97] Kerur N. Hirano Y. Tarallo V. Fowler B. J. Bastos-Carvalho A. Yasuma T. (2013). TLR-independent and P2X7-dependent signaling mediate Alu RNA-induced NLRP3 inflammasome activation in geographic atrophy. *Invest. Ophthalmol. Vis. Sci.* 54 7395–7401. 10.1167/iovs.13-12500 24114535 PMC3825570

[B98] Khadra A. Tomic M. Yan Z. Zemkova H. Sherman A. Stojilkovic S. S. (2013). Dual gating mechanism and function of P2X7 receptor channels. *Biophys. J.* 104 2612–2621. 10.1016/j.bpj.2013.05.006 23790369 PMC3686336

[B99] Kim J. E. Kang T. C. (2011). The P2X7 receptor-pannexin-1 complex decreases muscarinic acetylcholine receptor-mediated seizure susceptibility in mice. *J. Clin. Invest.* 121 2037–2047. 10.1172/JCI44818 21505260 PMC3083785

[B100] Koch-Nolte F. Eichhoff A. Pinto-Espinoza C. Schwarz N. Schafer T. Menzel S. (2019). Novel biologics targeting the P2X7 ion channel. *Curr. Opin. Pharmacol.* 47 110–118. 10.1016/j.coph.2019.03.001 30986625

[B101] Kong Q. Wang M. Liao Z. Camden J. M. Yu S. Simonyi A. (2005). P2X(7) nucleotide receptors mediate caspase-8/9/3-dependent apoptosis in rat primary cortical neurons. *Purinergic Signal.* 1 337–347. 10.1007/s11302-005-7145-5 18404518 PMC2096553

[B102] Kunkle B. W. Grenier-Boley B. Sims R. Bis J. C. Damotte V. Naj A. C. (2019). Genetic meta-analysis of diagnosed Alzheimer’s disease identifies new risk loci and implicates Abeta, tau, immunity and lipid processing. *Nat. Genet.* 51 414–430. 10.1038/s41588-019-0358-2 30820047 PMC6463297

[B103] Lambert J. C. Heath S. Even G. Campion D. Sleegers K. Hiltunen M. (2009). Genome-wide association study identifies variants at CLU and CR1 associated with Alzheimer’s disease. *Nat. Genet.* 41 1094–1099. 10.1038/ng.439 19734903

[B104] Lambert J. C. Ibrahim-Verbaas C. A. Harold D. Naj A. C. Sims R. Bellenguez C. (2013). Meta-analysis of 74,046 individuals identifies 11 new susceptibility loci for Alzheimer’s disease. *Nat. Genet.* 45 1452–1458. 10.1038/ng.2802 24162737 PMC3896259

[B105] Langer H. F. Choi E. Y. Zhou H. Schleicher R. Chung K. J. Tang Z. (2012). Platelets contribute to the pathogenesis of experimental autoimmune encephalomyelitis. *Circ. Res.* 110 1202–1210. 10.1161/CIRCRESAHA.111.256370 22456181 PMC3382058

[B106] Le Feuvre R. A. Brough D. Touzani O. Rothwell N. J. (2003). Role of P2X7 receptors in ischemic and excitotoxic brain injury *in vivo*. *J. Cereb. Blood Flow Metab.* 23 381–384. 10.1097/00004647-200303000-00013 12621313

[B107] Leeson H. C. Kasherman M. A. Chan-Ling T. Lovelace M. D. Brownlie J. C. Toppinen K. M. (2018). P2X7 receptors regulate phagocytosis and proliferation in adult hippocampal and SVZ neural progenitor cells: implications for inflammation in neurogenesis. *Stem Cells* 36 1764–1777. 10.1002/stem.2894 30068016 PMC6635745

[B108] Leon-Otegui M. Gomez-Villafuertes R. Diaz-Hernandez J. I. Diaz-Hernandez M. Miras-Portugal M. T. Gualix J. (2011). Opposite effects of P2X7 and P2Y2 nucleotide receptors on alpha-secretase-dependent APP processing in Neuro-2a cells. *FEBS Lett.* 585 2255–2262. 10.1016/j.febslet.2011.05.048 21651910

[B109] Liu T. Yamaguchi Y. Shirasaki Y. Shikada K. Yamagishi M. Hoshino K. (2014). Single-cell imaging of caspase-1 dynamics reveals an all-or-none inflammasome signaling response. *Cell Rep.* 8 974–982. 10.1016/j.celrep.2014.07.012 25127135

[B110] Liu X. Zhang Z. Ruan J. Pan Y. Magupalli V. G. Wu H. (2016). Inflammasome-activated gasdermin D causes pyroptosis by forming membrane pores. *Nature* 535 153–158. 10.1038/nature18629 27383986 PMC5539988

[B111] Lopez-Castejon G. Brough D. (2011). Understanding the mechanism of IL-1beta secretion. *Cytokine Growth Factor Rev.* 22 189–195. 10.1016/j.cytogfr.2011.10.001 22019906 PMC3714593

[B112] Lorden G. Sanjuan-Garcia I. De Pablo N. Meana C. Alvarez-Miguel I. Perez-Garcia M. T. (2017). Lipin-2 regulates NLRP3 inflammasome by affecting P2X7 receptor activation. *J. Exp. Med.* 214 511–528. 10.1084/jem.20161452 28031477 PMC5294860

[B113] Lovelace M. D. Gu B. J. Eamegdool S. S. Weible M. W. II Wiley J. S. Allen D. G. (2015). P2X7 receptors mediate innate phagocytosis by human neural precursor cells and neuroblasts. *Stem Cells* 33 526–541. 10.1002/stem.1864 25336287

[B114] MacKenzie A. Wilson H. L. Kiss-Toth E. Dower S. K. North R. A. Surprenant A. (2001). Rapid secretion of interleukin-1beta by microvesicle shedding. *Immunity* 15 825–835. 10.1016/s1074-7613(01)00229-1 11728343

[B115] Mahaut-Smith M. P. Jones S. Evans R. J. (2011). The P2X1 receptor and platelet function. *Purinergic Signal.* 7 341–356. 10.1007/s11302-011-9224-0 21484087 PMC3166991

[B116] Marchini J. Donnelly P. Cardon L. R. (2005). Genome-wide strategies for detecting multiple loci that influence complex diseases. *Nat. Genet.* 37 413–417. 10.1038/ng1537 15793588

[B117] Marioni R. E. Harris S. E. Zhang Q. Mcrae A. F. Hagenaars S. P. Hill W. D. (2018). GWAS on family history of Alzheimer’s disease. *Transl. Psychiatry* 8:99. 10.1038/s41398-018-0150-6 PMC5959890 29777097

[B118] Martin E. Amar M. Dalle C. Youssef I. Boucher C. Le Duigou C. (2019). New role of P2X7 receptor in an Alzheimer’s disease mouse model. *Mol. Psychiatry* 24 108–125. 10.1038/s41380-018-0108-3 29934546 PMC6756107

[B119] Mascanfroni I. D. Takenaka M. C. Yeste A. Patel B. Wu Y. Kenison J. E. (2015). Metabolic control of type 1 regulatory T cell differentiation by AHR and HIF1-alpha. *Nat. Med.* 21 638–646. 10.1038/nm.3868 26005855 PMC4476246

[B120] Mattson M. P. (1997). Cellular actions of beta-amyloid precursor protein and its soluble and fibrillogenic derivatives. *Physiol. Rev.* 77 1081–1132. 10.1152/physrev.1997.77.4.1081 9354812

[B121] Matute C. Torre I. Perez-Cerda F. Perez-Samartin A. Alberdi E. Etxebarria E. (2007). P2X(7) receptor blockade prevents ATP excitotoxicity in oligodendrocytes and ameliorates experimental autoimmune encephalomyelitis. *J. Neurosci.* 27 9525–9533. 10.1523/jneurosci.0579-07.2007 17728465 PMC6673129

[B122] McLarnon J. G. Ryu J. K. Walker D. G. Choi H. B. (2006). Upregulated expression of purinergic P2X(7) receptor in Alzheimer disease and amyloid-beta peptide-treated microglia and in peptide-injected rat hippocampus. *J. Neuropathol. Exp. Neurol.* 65 1090–1097. 10.1097/01.jnen.0000240470.97295.d3 17086106

[B123] Mellouk A. Bobe P. (2019). CD8(+), but not CD4(+) effector/memory T cells, express the CD44(high)CD45RB(high) phenotype with aging, which displays reduced expression levels of P2X7 receptor and ATP-induced cellular responses. *FASEB J.* 33 3225–3236. 10.1096/fj.201800867r 30383448

[B124] Messemer N. Kunert C. Grohmann M. Sobottka H. Nieber K. Zimmermann H. (2013). P2X7 receptors at adult neural progenitor cells of the mouse subventricular zone. *Neuropharmacology* 73 122–137. 10.1016/j.neuropharm.2013.05.017 23727220

[B125] Metzger M. W. Walser S. M. Aprile-Garcia F. Dedic N. Chen A. Holsboer F. (2017). Genetically dissecting P2rx7 expression within the central nervous system using conditional humanized mice. *Purinergic Signal.* 13 153–170. 10.1007/s11302-016-9546-z 27858314 PMC5432476

[B126] Miras-Portugal M. T. Sebastian-Serrano A. De Diego Garcia L. Diaz-Hernandez M. (2017). Neuronal P2X7 receptor: involvement in neuronal physiology and pathology. *J. Neurosci.* 37 7063–7072. 10.1523/JNEUROSCI.3104-16.2017 28747389 PMC6705729

[B127] Mizutani T. Fowler B. J. Kim Y. Yasuma R. Krueger L. A. Gelfand B. D. (2015). Nucleoside reverse transcriptase inhibitors suppress laser-induced choroidal neovascularization in mice. *Invest. Ophthalmol. Vis. Sci.* 56 7122–7129. 10.1167/iovs.15-17440 26529046 PMC4634627

[B128] Monif M. Reid C. A. Powell K. L. Smart M. L. Williams D. A. (2009). The P2X7 receptor drives microglial activation and proliferation: a trophic role for P2X7R pore. *J. Neurosci.* 29 3781–3791. 10.1523/JNEUROSCI.5512-08.2009 19321774 PMC6665035

[B129] Morciano G. Sarti A. C. Marchi S. Missiroli S. Falzoni S. Raffaghello L. (2017). Use of luciferase probes to measure ATP in living cells and animals. *Nat. Protoc.* 12 1542–1562. 10.1038/nprot.2017.052 28683062

[B130] Naj A. C. Jun G. Beecham G. W. Wang L. S. Vardarajan B. N. Buros J. (2011). Common variants at MS4A4/MS4A6E, CD2AP, CD33 and EPHA1 are associated with late-onset Alzheimer’s disease. *Nat. Genet.* 43 436–441. 10.1038/ng.801 21460841 PMC3090745

[B131] Narcisse L. Scemes E. Zhao Y. Lee S. C. Brosnan C. F. (2005). The cytokine IL-1beta transiently enhances P2X7 receptor expression and function in human astrocytes. *Glia* 49 245–258. 10.1002/glia.20110 15472991 PMC2586293

[B132] Nicke A. Kuan Y. H. Masin M. Rettinger J. Marquez-Klaka B. Bender O. (2009). A functional P2X7 splice variant with an alternative transmembrane domain 1 escapes gene inactivation in P2X7 knock-out mice. *J. Biol. Chem.* 284 25813–25822. 10.1074/jbc.M109.033134 19546214 PMC2757983

[B133] Ousingsawat J. Wanitchakool P. Kmit A. Romao A. M. Jantarajit W. Schreiber R. (2015). Anoctamin 6 mediates effects essential for innate immunity downstream of P2X7 receptors in macrophages. *Nat. Commun.* 6:6245. 10.1038/ncomms7245 25651887

[B134] Panenka W. Jijon H. Herx L. M. Armstrong J. N. Feighan D. Wei T. (2001). P2X7-like receptor activation in astrocytes increases chemokine monocyte chemoattractant protein-1 expression via mitogen-activated protein kinase. *J. Neurosci.* 21 7135–7142. 10.1523/jneurosci.21-18-07135.2001 11549724 PMC6762971

[B135] Pelegrin P. Surprenant A. (2006). Pannexin-1 mediates large pore formation and interleukin-1beta release by the ATP-gated P2X7 receptor. *EMBO J.* 25 5071–5082. 10.1038/sj.emboj.7601378 17036048 PMC1630421

[B136] Peverini L. Beudez J. Dunning K. Chataigneau T. Grutter T. (2018). New insights into permeation of large cations through ATP-Gated P2X receptors. *Front. Mol. Neurosci.* 11:265. 10.3389/fnmol.2018.00265 30108481 PMC6080412

[B137] Pippel A. Stolz M. Woltersdorf R. Kless A. Schmalzing G. Markwardt F. (2017). Localization of the gate and selectivity filter of the full-length P2X7 receptor. *Proc. Natl. Acad. Sci. U.S.A.* 114 E2156–E2165. 10.1073/pnas.1610414114 28235784 PMC5358401

[B138] Qu Y. Dubyak G. R. (2009). P2X7 receptors regulate multiple types of membrane trafficking responses and non-classical secretion pathways. *Purinergic Signal.* 5 163–173. 10.1007/s11302-009-9132-8 19189228 PMC2686822

[B139] Qu Y. Franchi L. Nunez G. Dubyak G. R. (2007). Nonclassical IL-1 beta secretion stimulated by P2X7 receptors is dependent on inflammasome activation and correlated with exosome release in murine macrophages. *J. Immunol.* 179 1913–1925. 10.4049/jimmunol.179.3.1913 17641058

[B140] Qu Y. Misaghi S. Newton K. Gilmour L. L. Louie S. Cupp J. E. (2011). Pannexin-1 is required for ATP release during apoptosis but not for inflammasome activation. *J. Immunol.* 186 6553–6561. 10.4049/jimmunol.1100478 21508259

[B141] Ramanan V. K. Saykin A. J. (2013). Pathways to neurodegeneration: mechanistic insights from GWAS in Alzheimer’s disease, Parkinson’s disease, and related disorders. *Am. J. Neurodegener. Dis.* 2 145–175. 24093081 PMC3783830

[B142] Rampe D. Wang L. Ringheim G. E. (2004). P2X7 receptor modulation of beta-amyloid- and LPS-induced cytokine secretion from human macrophages and microglia. *J. Neuroimmunol.* 147 56–61. 10.1016/j.jneuroim.2003.10.014 14741428

[B143] Rassendren F. Audinat E. (2016). Purinergic signaling in epilepsy. *J. Neurosci. Res.* 94 781–793. 10.1002/jnr.23770 27302739

[B144] Riedel T. Schmalzing G. Markwardt F. (2007). Influence of extracellular monovalent cations on pore and gating properties of P2X7 receptor-operated single-channel currents. *Biophys. J.* 93 846–858. 10.1529/biophysj.106.103614 17483156 PMC1913143

[B145] Rigato C. Swinnen N. Buckinx R. Couillin I. Mangin J. M. Rigo J. M. (2012). Microglia proliferation is controlled by P2X7 receptors in a Pannexin-1-independent manner during early embryonic spinal cord invasion. *J. Neurosci.* 32 11559–11573. 10.1523/jneurosci.1042-12.2012 22915101 PMC6703767

[B146] Rissiek B. Haag F. Boyer O. Koch-Nolte F. Adriouch S. (2015). P2X7 on mouse T cells: one channel, many functions. *Front. Immunol.* 6:204. 10.3389/fimmu.2015.00204 26042119 PMC4436801

[B147] Robinson L. E. Shridar M. Smith P. Murrell-Lagnado R. D. (2014). Plasma membrane cholesterol as a regulator of human and rodent P2X7 receptor activation and sensitization. *J. Biol. Chem.* 289 31983–31994. 10.1074/jbc.M114.574699 25281740 PMC4231676

[B148] Rodrigues R. J. Tome A. R. Cunha R. A. (2015). ATP as a multi-target danger signal in the brain. *Front. Neurosci.* 9:148. 10.3389/fnins.2015.00148 25972780 PMC4412015

[B149] Rozmer K. Gao P. Araujo M. G. L. Khan M. T. Liu J. Rong W. (2017). Pilocarpine-induced status epilepticus increases the sensitivity of P2X7 and P2Y1 receptors to nucleotides at neural progenitor cells of the juvenile rodent hippocampus. *Cereb. Cortex* 27 3568–3585. 10.1093/cercor/bhw178 27341850

[B150] Ruhl S. Shkarina K. Demarco B. Heilig R. Santos J. C. Broz P. (2018). ESCRT-dependent membrane repair negatively regulates pyroptosis downstream of GSDMD activation. *Science* 362 956–960. 10.1126/science.aar7607 30467171

[B151] Russi A. E. Walker-Caulfield M. E. Guo Y. Lucchinetti C. F. Brown M. A. (2016). Meningeal mast cell-T cell crosstalk regulates T cell encephalitogenicity. *J. Autoimmun.* 73 100–110. 10.1016/j.jaut.2016.06.015 27396526 PMC6364701

[B152] Sabelko-Downes K. A. Russell J. H. Cross A. H. (1999). Role of Fas–FasL interactions in the pathogenesis and regulation of autoimmune demyelinating disease. *J. Neuroimmunol.* 100 42–52. 10.1016/s0165-5728(99)00191-5 10695714

[B153] Sadovnick A. D. Gu B. J. Traboulsee A. L. Bernales C. Q. Encarnacion M. Yee I. M. (2017). Purinergic receptors P2RX4 and P2RX7 in familial multiple sclerosis. *Hum. Mutat.* 38 736–744. 10.1002/humu.23218 28326637 PMC5429140

[B154] Safya H. Mellouk A. Legrand J. Le Gall S. M. Benbijja M. Kanellopoulos-Langevin C. (2018). Variations in cellular responses of mouse T cells to adenosine-5’-triphosphate stimulation do not depend on P2X7 receptor expression levels but on their activation and differentiation stage. *Front. Immunol.* 9:360. 10.3389/fimmu.2018.00360 29535730 PMC5835135

[B155] Sanz J. M. Chiozzi P. Ferrari D. Colaianna M. Idzko M. Falzoni S. (2009). Activation of microglia by amyloid {beta} requires P2X7 receptor expression. *J. Immunol.* 182 4378–4385. 10.4049/jimmunol.0803612 19299738

[B156] Savio L. E. B. De Andrade Mello P. Da Silva C. G. Coutinho-Silva R. (2018). The P2X7 receptor in inflammatory diseases: angel or demon? *Front. Pharmacol.* 9:52. 10.3389/fphar.2018.00052 29467654 PMC5808178

[B157] Sborgi L. Ruhl S. Mulvihill E. Pipercevic J. Heilig R. Stahlberg H. (2016). GSDMD membrane pore formation constitutes the mechanism of pyroptotic cell death. *EMBO J.* 35 1766–1778. 10.15252/embj.201694696 27418190 PMC5010048

[B158] Schachter J. Motta A. P. De Souza Zamorano A. Da Silva-Souza H. A. Guimaraes M. Z. Persechini P. M. (2008). ATP-induced P2X7-associated uptake of large molecules involves distinct mechanisms for cations and anions in macrophages. *J. Cell Sci.* 121 3261–3270. 10.1242/jcs.029991 18782864

[B159] Schmid-Burgk J. L. Chauhan D. Schmidt T. Ebert T. S. Reinhardt J. Endl E. (2016). A Genome-wide CRISPR (Clustered Regularly Interspaced Short Palindromic Repeats) screen identifies NEK7 as an essential component of NLRP3 inflammasome activation. *J. Biol. Chem.* 291 103–109. 10.1074/jbc.C115.700492 26553871 PMC4697147

[B160] Secor V. H. Secor W. E. Gutekunst C. A. Brown M. A. (2000). Mast cells are essential for early onset and severe disease in a murine model of multiple sclerosis. *J. Exp. Med.* 191 813–822. 10.1084/jem.191.5.813 10704463 PMC2195850

[B161] Seman M. Adriouch S. Scheuplein F. Krebs C. Freese D. Glowacki G. (2003). NAD-induced T cell death: ADP-ribosylation of cell surface proteins by ART2 activates the cytolytic P2X7 purinoceptor. *Immunity* 19 571–582. 14563321 10.1016/s1074-7613(03)00266-8

[B162] Sharif H. Wang L. Wang W. L. Magupalli V. G. Andreeva L. Qiao Q. (2019). Structural mechanism for NEK7-licensed activation of NLRP3 inflammasome. *Nature* 570 338–343. 10.1038/s41586-019-1295-z 31189953 PMC6774351

[B163] Sharp A. J. Polak P. E. Simonini V. Lin S. X. Richardson J. C. Bongarzone E. R. (2008). P2x7 deficiency suppresses development of experimental autoimmune encephalomyelitis. *J. Neuroinflammation* 5:33. 10.1186/1742-2094-5-33 18691411 PMC2518548

[B164] Shi H. Wang Y. Li X. Zhan X. Tang M. Fina M. (2016). NLRP3 activation and mitosis are mutually exclusive events coordinated by NEK7, a new inflammasome component. *Nat. Immunol.* 17 250–258. 10.1038/ni.3333 26642356 PMC4862588

[B165] Shi J. Zhao Y. Wang K. Shi X. Wang Y. Huang H. (2015). Cleavage of GSDMD by inflammatory caspases determines pyroptotic cell death. *Nature* 526 660–665. 10.1038/nature15514 26375003

[B166] Skaper S. D. Facci L. Culbert A. A. Evans N. A. Chessell I. Davis J. B. (2006). P2X(7) receptors on microglial cells mediate injury to cortical neurons *in vitro*. *Glia* 54 234–242. 10.1002/glia.20379 16817206

[B167] Solle M. Labasi J. Perregaux D. G. Stam E. Petrushova N. Koller B. H. (2001). Altered cytokine production in mice lacking P2X(7) receptors. *J. Biol. Chem.* 276 125–132. 11016935 10.1074/jbc.M006781200

[B168] Sonia D. Souza C. Li Z. Luke Maxwell D. Trusler O. Murphy M. (2018). Platelets drive inflammation and target gray matter and the retina in autoimmune-mediated encephalomyelitis. *J. Neuropathol. Exp. Neurol.* 77 567–576. 10.1093/jnen/nly032 29757405

[B169] Starossom S. C. Veremeyko T. Yung A. W. Dukhinova M. Au C. Lau A. Y. (2015). Platelets play differential role during the initiation and progression of autoimmune neuroinflammation. *Circ. Res.* 117 779–792. 10.1161/CIRCRESAHA.115.306847 26294656 PMC4716010

[B170] Steinberg T. H. Newman A. S. Swanson J. A. Silverstein S. C. (1987). ATP4- permeabilizes the plasma membrane of mouse macrophages to fluorescent dyes. *J. Biol. Chem.* 262 8884–8888. 3597398

[B171] Tang T. Lang X. Xu C. Wang X. Gong T. Yang Y. (2017). CLICs-dependent chloride efflux is an essential and proximal upstream event for NLRP3 inflammasome activation. *Nat. Commun.* 8:202. 10.1038/s41467-017-00227-x 28779175 PMC5544706

[B172] Taylor J. P. Brown R. H. Jr. Cleveland D. W. (2016). Decoding ALS: from genes to mechanism. *Nature* 539 197–206. 10.1038/nature20413 27830784 PMC5585017

[B173] Taylor S. R. Gonzalez-Begne M. Sojka D. K. Richardson J. C. Sheardown S. A. Harrison S. M. (2009). Lymphocytes from P2X7-deficient mice exhibit enhanced P2X7 responses. *J. Leukoc. Biol.* 85 978–986. 10.1189/jlb.0408251 19276178 PMC2698584

[B174] Tsao H. K. Chiu P. H. Sun S. H. (2013). PKC-dependent ERK phosphorylation is essential for P2X7 receptor-mediated neuronal differentiation of neural progenitor cells. *Cell Death Dis.* 4:e751. 10.1038/cddis.2013.274 23907465 PMC3763436

[B175] Vessey K. A. Gu B. J. Jobling A. I. Phipps J. A. Greferath U. Tran M. X. (2017). Loss of function of P2X7 receptor scavenger activity in aging mice: a novel model for investigating the early pathogenesis of age-related macular degeneration. *Am. J. Pathol.* 187 1670–1685. 10.1016/j.ajpath.2017.04.016 28628761

[B176] Witting A. Chen L. Cudaback E. Straiker A. Walter L. Rickman B. (2006). Experimental autoimmune encephalomyelitis disrupts endocannabinoid-mediated neuroprotection. *Proc. Natl. Acad. Sci. U.S.A.* 103 6362–6367. 10.1073/pnas.0510418103 16571660 PMC1458883

[B177] Witting A. Walter L. Wacker J. Moller T. Stella N. (2004). P2X7 receptors control 2-arachidonoylglycerol production by microglial cells. *Proc. Natl. Acad. Sci. U.S.A.* 101 3214–3219. 10.1073/pnas.0306707101 14976257 PMC365769

[B178] Yiangou Y. Facer P. Durrenberger P. Chessell I. P. Naylor A. Bountra C. (2006). COX-2, CB2 and P2X7-immunoreactivities are increased in activated microglial cells/macrophages of multiple sclerosis and amyotrophic lateral sclerosis spinal cord. *BMC Neurol.* 6:12. 10.1186/1471-2377-6-12 16512913 PMC1413551

[B179] Yip L. Woehrle T. Corriden R. Hirsh M. Chen Y. Inoue Y. (2009). Autocrine regulation of T-cell activation by ATP release and P2X7 receptors. *FASEB J.* 23 1685–1693. 10.1096/fj.08-126458 19211924 PMC2718802

[B180] Young C. N. J. Gorecki D. C. (2018). P2RX7 purinoceptor as a therapeutic target-the second coming? *Front. Chem.* 6:248. 10.3389/fchem.2018.00248 30003075 PMC6032550

[B181] Zanoni I. Tan Y. Di Gioia M. Springstead J. R. Kagan J. C. (2017). By capturing inflammatory lipids released from dying cells, the receptor CD14 induces inflammasome-dependent phagocyte hyperactivation. *Immunity* 47 697–709.e3. 10.1016/j.immuni.2017.09.010 29045901 PMC5747599

[B182] Zhou R. Tardivel A. Thorens B. Choi I. Tschopp J. (2010). Thioredoxin-interacting protein links oxidative stress to inflammasome activation. *Nat. Immunol.* 11 136–140. 10.1038/ni.1831 20023662

